# Glacier ice archives nearly 15,000-year-old microbes and phages

**DOI:** 10.1186/s40168-021-01106-w

**Published:** 2021-07-20

**Authors:** Zhi-Ping Zhong, Funing Tian, Simon Roux, M. Consuelo Gazitúa, Natalie E. Solonenko, Yueh-Fen Li, Mary E. Davis, James L. Van Etten, Ellen Mosley-Thompson, Virginia I. Rich, Matthew B. Sullivan, Lonnie G. Thompson

**Affiliations:** 1 Byrd Polar and Climate Research Center, Ohio State University, Columbus, OH USA; 2 Department of Microbiology, Ohio State University, Columbus, OH USA; 3 Center of Microbiome Science, Ohio State University, Columbus, OH USA; 4 Department of Energy Joint Genome Institute, Lawrence Berkeley National Laboratory, Berkeley, CA USA; 5 Department of Plant Pathology and Nebraska Center for Virology, University of Nebraska–Lincoln, Lincoln, NE USA; 6 Department of Geography, Ohio State University, Columbus, OH USA; 7 Department of Civil, Environmental and Geodetic Engineering, Ohio State University, Columbus, OH USA; 8 School of Earth Sciences, Ohio State University, Columbus, OH USA

**Keywords:** Guliya ice cap, Mountain glacier ice, Surface decontamination, Ice microbes, Ice viruses, *Methylobacterium*, *Sphingomonas*, *Janthinobacterium*

## Abstract

**Background:**

Glacier ice archives information, including microbiology, that helps reveal paleoclimate histories and predict future climate change. Though glacier-ice microbes are studied using culture or amplicon approaches, more challenging metagenomic approaches, which provide access to functional, genome-resolved information and viruses, are under-utilized, partly due to low biomass and potential contamination.

**Results:**

We expand existing clean sampling procedures using controlled artificial ice-core experiments and adapted previously established low-biomass metagenomic approaches to study glacier-ice viruses. Controlled sampling experiments drastically reduced mock contaminants including bacteria, viruses, and free DNA to background levels. Amplicon sequencing from eight depths of two Tibetan Plateau ice cores revealed common glacier-ice lineages including *Janthinobacterium*, *Polaromonas*, *Herminiimonas*, *Flavobacterium*, *Sphingomonas*, and *Methylobacterium* as the dominant genera, while microbial communities were significantly different between two ice cores, associating with different climate conditions during deposition. Separately, ~355- and ~14,400-year-old ice were subject to viral enrichment and low-input quantitative sequencing, yielding genomic sequences for 33 vOTUs. These were virtually all unique to this study, representing 28 novel genera and not a single species shared with 225 environmentally diverse viromes. Further, 42.4% of the vOTUs were identifiable temperate, which is significantly higher than that in gut, soil, and marine viromes, and indicates that temperate phages are possibly favored in glacier-ice environments before being frozen. In silico host predictions linked 18 vOTUs to co-occurring abundant bacteria (*Methylobacterium*, *Sphingomonas*, and *Janthinobacterium*), indicating that these phages infected ice-abundant bacterial groups before being archived. Functional genome annotation revealed four virus-encoded auxiliary metabolic genes, particularly two motility genes suggest viruses potentially facilitate nutrient acquisition for their hosts. Finally, given their possible importance to methane cycling in ice, we focused on *Methylobacterium* viruses by contextualizing our ice-observed viruses against 123 viromes and prophages extracted from 131 *Methylobacterium* genomes, revealing that the archived viruses might originate from soil or plants.

**Conclusions:**

Together, these efforts further microbial and viral sampling procedures for glacier ice and provide a first window into viral communities and functions in ancient glacier environments. Such methods and datasets can potentially enable researchers to contextualize new discoveries and begin to incorporate glacier-ice microbes and their viruses relative to past and present climate change in geographically diverse regions globally.

Video Abstract

**Supplementary Information:**

The online version contains supplementary material available at 10.1186/s40168-021-01106-w.

## Background

The first reports of microbes in glacier ice appeared early in the twentieth century [1] but were largely ignored until the 1980s when microbes were investigated in the deep Vostok ice core [2] and subsequent studies near the end of the 1990s (reviewed in [3–6]). These studies revealed microbial cell concentrations of 10^2^ to 10^4^ cells ml^−1^ in most glacier-ice samples [4], which are several orders of magnitude lower than other environments such as seawater or soils [7]. The microbes identified in glacier cores potentially represent the microbes in the atmosphere at the time of their deposition [3, 8], though we cannot rule out post-deposition metabolisms of microbes [9]. Microbial communities of glacier cores were reported to correlate with variations in dust and ion concentrations [10–14]. A long temporal record (27k to 9.6k years before present) of prokaryotic cell concentration from a deep West Antarctic ice core revealed that airborne prokaryotic cell deposition differed during the Last Glacial Maximum, Last Deglaciation, and Early Holocene periods [8]. Hence, the glacier-ice microbes may reflect climatic and environmental conditions during that time of deposition [3]. Taxonomically, *Proteobacteria*, *Actinobacteria*, *Firmicutes*, and *Bacteroidetes* are the dominant bacterial phyla found in ice cores [4, 15–17]. Bacteria of above phyla have been successfully cultured from very old frozen glacier ice [18–21], including some that were believed to have been preserved for >750,000 years [19] because of the subzero temperatures and low water activities within the ice matrix. Some bacteria were preserved as spores in glacier ice [22, 23]. Although there is currently no direct evidence for in situ activity, some studies have hinted at the possibility of microbial activity in frozen glacier ice based on the detection of some excess gases (e.g., CO_2_, CH_4_, and N_2_O), which may be produced by post-depositional microbial metabolism [24–26].

Though most ice core microbiological studies have focused on microbial communities using culture-dependent and culture-independent (e.g., 16S rRNA gene amplicon sequencing) methods, and how to use them to understand past climatic and environmental conditions archived in the glaciers [3–6], there have been only two reports of viruses in ancient glacier ice. One detected the atmosphere-originated tomato mosaic tobamovirus RNA in a 140,000-year-old Greenland ice core using reverse-transcription PCR amplification [27], and the other reported the presence of virus-like particles (VLPs) deep (i.e., 2749- and 3556-m depth) in the Vostok ice core using transmission electron microscopy [3]. Ancient viruses were also reported from other environments such as permafrost [28] and frozen animal feces [29]. The viral abundance was reported to range from 0.1 to 5.6 × 10^5^ VLPs ml^−1^ in the surface ice (top 90 cm) of two Arctic glaciers in Svalbard [30], while the cryoconite holes on the surface of some glaciers possess abundant and active viral communities [30–32]. For example, 10^8^ to 10^9^ VLPs g^−1^ of sediment and viral production rate of 10^7^ to 10^8^ VLPs g^−1^ h^−1^ were detected in Arctic cryoconite holes [31]. However, virtually, nothing is known about the archived ancient glacier-ice viral genomes or communities, which might be active on the glacier surfaces before being frozen tens of thousands of years ago. If other microbial ecosystems are any indication, this likely provides hints for potentially major players in these archived communities before being frozen. For example, in marine systems, viruses are abundant (10^6^ to 10^9^ particles ml^−1^ of seawater [33]), with virulent viruses altering microbial communities through lysis, horizontal gene transfer, and metabolic reprogramming (e.g., [34–38]), and temperate viruses modulating host gene regulation and providing novel niche-defining features [39]. In the cryosphere, viruses are much less known, but some data are starting to emerge, such as the studies of viral ecology and evolution in Arctic cryoconite holes [40, 41] and a recent work in Arctic sea ice and ancient cryopegs which revealed viruses are abundant, predicted to infect dominant microbial community members, and encoded auxiliary metabolic genes that enabled host adaptations to extreme cold and salt conditions [42]. Thus, even in these extreme conditions, it appears viruses can play key roles in the ecosystem when they and their hosts are active.

Problematically, beyond the expeditionary efforts required to obtain glacier ice cores, community metagenomics approaches are challenged by the low biomass of these samples. *First*, the low quantity of nucleic acids that can be extracted has left such samples intractable for methods that commonly require micrograms of nucleic acids for metagenomes. *Second*, because of low biomass, contamination from sampling, storage, and processing is a major issue as genetic material from contaminant organisms can muddle with and overwhelm material from the real glacier-ice community [43, 44]. For the former regarding low quantity of nucleic acids, significant progress has been made in seawater viral communities both in ultra-low input sample preparation [45–48], data interpretation, and standards [36, 49, 50]. For the latter, clean sampling techniques and surface decontamination strategies have been pioneered to remove potential contaminants on ice core surfaces before melting them for microbial analysis [51–54]. In addition, background controls were processed in parallel to authentic ice samples to track and in silico remove suspected contaminants introduced during the processing of ice in the laboratory [17, 22, 53]. We acknowledge that these available methods are not perfect and may still have limitations in decontamination, e.g., it is hard, if not impossible, to demonstrate the removal of all “contaminants” by these methods, while these are the best methods available to date for efficiently eliminating the suspected microbial contaminants and have been adopted for many microbial investigations of glacier ice (e.g., [14, 17, 22, 25]). However, the removal efficiency of viral “contaminants” is yet evaluated on the ice core surface.

Here, we sought to apply these available approaches, including the low-biomass metagenomics approaches initially developed from seawater and the decontamination techniques, to glacier ice, and further establish clean procedures to remove microbial and viral contaminants on ice surfaces through artificial-ice-core “contamination” experiments. Once optimized, we applied these updated procedures to investigate microbial and viral communities archived in two ice cores drilled on the summit (6710 m asl) and plateau (6200 m asl) of the Guliya ice cap (35.25°N; 81.48°E) in far northwestern Tibetan Plateau.

## Results and discussion

### Establishing clean surface-decontamination procedures with mock contaminants

**Fig. 1:**
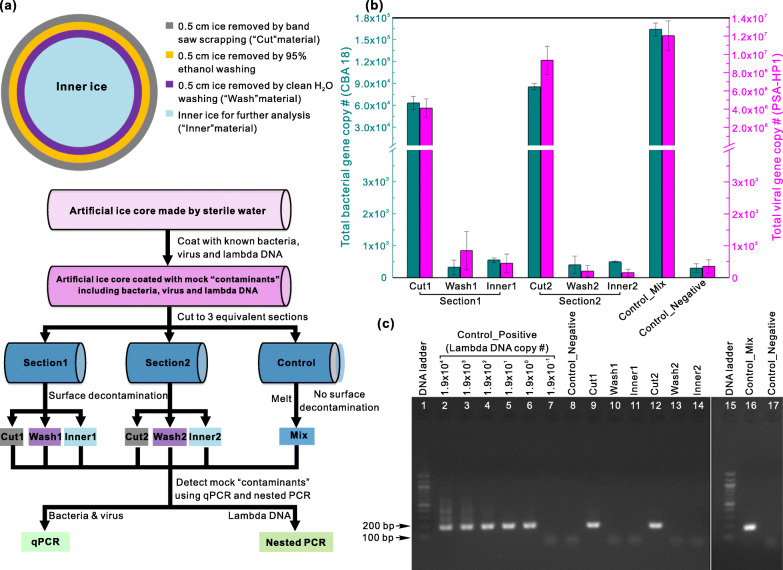
Establishment of decontamination protocol. **a** Schematic of layered removal of the outer core surface to obtain clean inner ice (top panel) and experimental approach to establish decontamination procedures using sterile artificial ice core sections coated with mock “contaminants” (down panel). Cut, wash, and inner represent ice samples collected from band saw scrapping, water washing, and the inner ice, respectively. Mix represents a sample from the melted ice of a control ice core section prepared without decontamination processing. The mock contaminants were detected by qPCR and nested PCR (see “Materials and methods”) in (**b**) and (**c**). **b** Total bacterial (dark teal color) and viral (purple color) numbers were quantified by qPCR using strain-designed primers in all samples collected in (**a**). **c** Lambda DNA was detected using nested PCR with designed outer and inner primer sets for lambda DNA. PCR products from inner primer sets were visualized by agarose gel electrophoresis; 1, 100bp DNA ladder; 2–7 represent 1.9×10^4^, 10^3^, 10^2^, 10^1^, 10^0^, and 10^−1^ (10-times dilution from standards) copies of lambda DNA, respectively, used as templates for nested PCR; 8, Control_Negative (no template); 9, Sample Cut1; 10, Wash1;11, Inner1; 12, Cut2; 13, Wash2; 14, Inner2; 15, 100bp DNA ladder (same as 1); 16, Control_Mix; 17, Control_Negative (same as 8)

In the field, no special procedures were used to avoid microbial contamination during ice core drilling, handling, and transport. Therefore, ice core surfaces likely contained microbial contaminants that impeded the identification of microbial communities archived in the ice [52, 55]. To develop a clean surface-decontamination procedure for removing possible microbial contaminants on the ice core surfaces and for collecting clean ice for microbial investigations, we constructed sterile artificial ice core sections and covered them with a known bacterium (*Cellulophaga baltica* strain 18, CBA 18), a known virus (*Pseudoalteromonas* phage PSA-HP1), and free DNA (from lambda phage), according to established protocols [52] (see “Materials and methods” and Fig. 1a). The decontamination procedure involved three sequential steps to remove a total of ~1.5 cm of the core radius, and the decontamination efficiency was evaluated (see “Materials and methods” and Fig. 1a).

The bacterial and viral contamination in each sample was quantified using strain-specific primers and qPCR (see “Materials and methods”). The contaminant bacteria and viruses were reduced by several orders of magnitude to background levels (Fig. 1b), after being processed with the surface-decontamination procedures described above (Fig. 1a and Additional file 2: Fig. S1). Even with extremely sensitive method (nested PCR), contaminant lambda phage DNA was not detected in the resulting inner ice (Fig. 1c). These results indicate that the decontamination procedure removed contaminants such as bacteria, viruses, and free DNA from the surface ice and left clean inner ice that was free of detectable contaminants for microbial and viral analysis. Earlier studies [51–54] have put foundational efforts to establish clean ice methods to decontaminate microbes; here, we constructed different decontamination systems (e.g., different washing facilities with three sequential steps; Additional file 2: Fig. S1) and expanded the clean procedures to also decontaminate viruses from glacier ice core surfaces.

### Decontamination method provides clean ice from glacier core sections

**Fig. 2:**
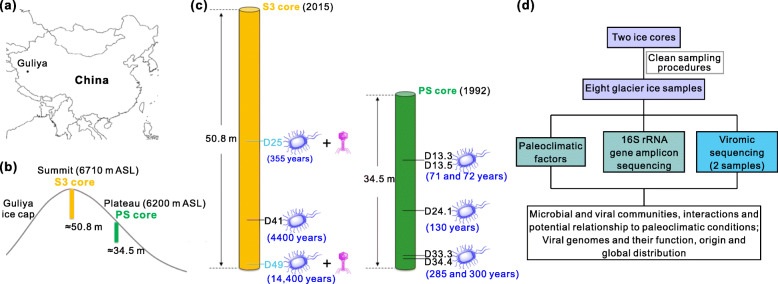
Sampling sites of glacier ice and an overview of experimental design. **a** Location of the Guliya ice cap; **b** drilling sites of the S3 and PS ice cores in Guliya ice cap; **c** sampling depths of eight ice samples used to investigate the microbial and viral communities; and **d** an overview of experimental design for microbial and viral investigations of collected ice samples. S3 and PS cores were drilled from the summit and plateau of Guliya ice cap, respectively (**b**). The drill date and length of the two ice cores and the approximate age of each sample are indicated (**c**). The sample names are coded by depth, e.g., for D13.3 is from 13.3 m below the glacier surface. All samples were subjected to microbial investigations, and two samples D25 and D49 (light blue) were selected for viral investigation

After we established that the surface-decontamination procedure removed surface contaminants, we then used authentic ice core sections to further evaluate the procedure. Two sections (samples D13.3 and D13.5, from 13.34 to 13.50 and 13.50 to 13.67 m depth, respectively) obtained from a plateau shallow ice core (PS ice core) drilled in 1992 from the plateau of the Guliya ice cap (Fig. 2a, b, c) were decontaminated using the procedures described above (Fig. 1a). The ice removed during saw cutting and water washing steps (cut: saw-scraped ice; wash: H_2_O-washed ice), along with the inner ice (inner) for each section, was collected as described above (Fig. 1a). Microbial profiles of six samples (three samples—cut, wash, and inner—from each of the two ice sections) were examined using Illumina Miseq 16S rRNA gene amplicon sequencing.

The 30 most abundant bacterial genera, each accounting for ≥0.5% of the sequences in at least one sample, comprised 94.7% of the total 72,000 sequences in the six samples (12,000 sequences each sample). These groups were designed as “major genera” and were selected to compare the microbial communities of all cut, wash, and inner samples for both ice sections (Additional file 2: Fig. S2A). Within each ice section, the most abundant genera were shared across the cut, wash, and inner samples (Additional file 2: Fig. S2A). For example, the 11 most abundant genera (i.e., an unclassified genus within *Microbacteriaceae*, an unclassified genus within *Comamonadaceae*, *Flavobacterium*, *Hymenobacter*, an unclassified genus within *Sphingobacteriaceae*, an unclassified genus within *Sporichthyaceae*, *Polaromonas*, an unclassified genus within *Actinomycetales*, *Nocardioides*, *Janthinobacterium*, and an unclassified genus within *Rhizobiales*; ordered by relative abundance) were represented in all three (i.e., inner, wash, and cut) D13.3 samples; these genera comprised 93.4%, 92.8%, and 89.1% of the microbial communities in the inner, wash, and cut samples, respectively (Additional file 2: Fig. S2A). In addition, results from a two-tailed paired *t*-test showed that the microbial communities did not change significantly across inner, wash, and cut samples of the same ice section (p values were 0.70–0.96 for all pairs of samples, i.e., cut versus wash, cut versus inner, and wash versus inner of each section). To further evaluate these results, we next compared the microbial communities at species level using the most abundant OTUs (n = 33), each of which accounted for ≥1.0% of the sequences in at least one sample. The summed relative abundance of these OTUs ranged from 71.6 to 78.6% in these samples (Additional file 1: Table S1). Similar to the comparisons at genus level, the inner, wash, and cut samples of the same ice section shared most of the top abundant OTUs (Additional file 2: Fig. S2b). Specially, 29 of 31 and 29 of 32 OTUs were shared between the inner and the other two removed ice samples (i.e., cut and wash) for the D13.3 and D13.5, respectively. These comparisons at both genus and species levels suggest that the contaminants on the ice core surface were not abundant and diverse enough to alter the overall microbial community composition of glacier ice based on the most abundant microbial groups in these ice core sections. Notably, the PS ice core was drilled in 1992 using an electromechanical drill with no drilling fluid [56]; in general, the surfaces of these cores are less contaminated than ice cores extracted using a fluid in the borehole [55].

Several OTUs were unique in the removed samples, including one OTU belonging to the genus *Acinetobacter* for sample D13.3, as well as two OTUs within the genus *Hymenobacter* and one unclassified bacterial OTU for sample D13.5 (Additional file 2: Table S1). We posit that these OTUs (<1.0%) might be contaminants removed from the ice core surface. We also note that there may also be natural variations in microbial communities across the same cross section of an ice core (here they were represented by cut, wash, and inner samples from the same depth), as uneven horizontal distribution of dust, nutrients, and microbes in an ice core is not unexpected and may reflect variation in deposition.

### Microbial profiles potentially differ between the PS and S3 ice cores

Once a clean decontamination procedure was established with both artificial ice cores and authentic ice core sections, we investigated the microbial and viral communities of two ice cores from Guliya ice cap (Fig. 2a, b, c, d). We first focused on microbial communities from five different depths (i.e., 13.3, 13.5, 24.1, 33.3, and 34.4 m) in the 1992 PS ice core, and compared them with the communities of three samples (i.e., D25, D41, and D49) from the 2015 summit core 3 (S3) (Fig. 2a, b, c, d). These three S3 samples were processed at the same time, and the 16S rRNA gene data for two (i.e., D41 and D49) of them were published previously to establish in silico decontamination method [17] and were cited in this study for comparison of microbial communities across eight depths of two ice cores from the same glacier. Four background controls were co-processed with the glacier ice samples to trace the background microbial profiles, which were then proportionally removed in silico from amplicon data of the ice core samples (see “Materials and methods”), according to our previously published method [17].

**Fig. 3:**
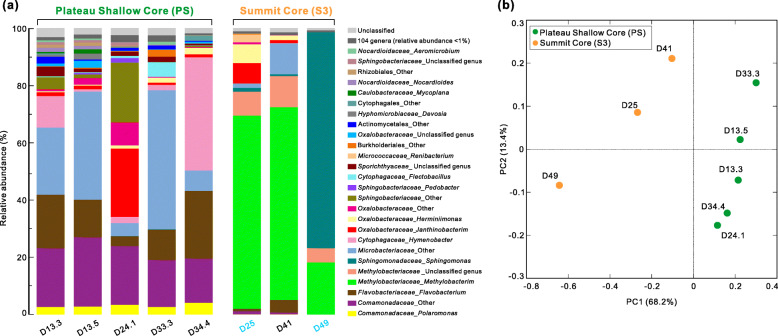
Distinct microbial profiles between PS and S3 ice cores. **a** Microbial profiles of the 26 most abundant genera in PS and S3 ice core samples. Profiles are illustrated as a percent of the total 16S rRNA gene amplicon sequences. The key indicates genera, preceded by family, or order in cases where family is not assigned. Genera labeled “Other” represent sequences with unknown genus-level taxonomy, i.e., distinct from taxonomically assigned genera in the reference database. The 26 most abundant genera, defined as those comprising at least 1.0% of the sequences in at least one ice sample, collectively represented >95.1% of each community. The total relative abundance of these genera was normalized to 100%. **b** PCoA showing sample clustering based on microbial communities at OTU (~species, 97% identity) level. Samples from the same ice core are marked with the same color. Sample names are indicated next to each symbol. PCoA was performed on the weighted UniFrac metric, which accounts for the relative abundance and inferred relatedness of the lineages present

After in silico decontamination, we compared the microbial community composition at genus level between and within ice cores. Reads were rarefied to 24,000 sequences in each sample, and collectively, the samples contained 254 bacterial genera, 118 of which were taxonomically identified to the genus level (Additional file 1: Table S2). The 26 most abundant genera, defined as those comprising at least 1.0% of sequences in at least one ice sample, represented >95.1% of each community (Fig. 3a). Bacterial genera including *Janthinobacterium* (relative abundance 1.0–23.8%), *Polaromonas* (2.6–4.1%), *Flavobacterium* (2.3–23.6%), and unknown genera within the families *Comamonadaceae* (15.5–24.3%) and *Microbacteriaceae* (7.1–48.5%) were abundant and present in all five PS samples (Fig. 3a). This indicates that members belonging to these lineages subsist over long periods of time in the environments before being frozen permanently, although their relative abundances vary across ice core depths (ages). These genera and families have also been reported as abundant groups in glacier ice cores by many previous studies (e.g., [4, 15, 17, 57–59]). The detection of bacterial sequences belonging to similar genera in ice core samples from different glaciers located around the world can be explained by the ubiquitous distribution of certain species in geographically distant environments [60]. The S3 and PS ice core samples shared some abundant genera, such as *Janthinobacterium*, *Herminiimonas*, and *Flavobacterium* (Fig. 3a); however, several abundant genera in the S3 samples were nearly absent in the PS samples, including *Sphingomonas*, *Methylobacterium*, and an unclassified genus in the family *Methylobacteriaceae* (Fig. 3a). Thus, there are potential differences in the microbial communities between the ice cores retrieved from the plateau (shallow part) and the summit of the Guliya ice cap.

We next used Principal Coordinates Analysis (PCoA) to compare microbial community compositions at OTU (~species; 97% identity) level among all eight samples and found that the communities clustered primarily by ice core (Fig. 3b), separating along the first principle coordinate (which accounted for 68.2% of community variability; the second axis accounted for 13.4%). Analysis of similarity statistics (ANOSIM) confirmed that the microbial communities of samples from the plateau core were significantly different from summit core samples (p = 0.02). Because of the differences in the elevation-relevant factors such as the wind power and temperature, the process from deposition to accumulation could be different between plateau and summit surfaces, which may further contribute to the variations in their microbial communities. In addition, all PS core samples were from the shallower part of the ice cap (top 34.5 m of the ~310-m thick ice field) [56] and were much younger than the three samples from the S3 core (~70–300 years versus ~355–14,400 years old; Additional file 1: Table S3), which were collected near the bottom of the summit ice core (~51-m length; Fig. 2). Therefore, the ice samples from the two different ice cores represent very different climate conditions at the time of deposition. This is further illustrated by variations in several environmental parameters (e.g., concentration of insoluble dust and ions such as sulfate and sodium) measured in the two ice cores (Additional file 1: Table S3). To further identify the environmental parameters potentially influencing these microbial communities, two-tailed Mantel tests were performed to examine the relationships between environmental properties (Additional file 1: Table S3) and microbial community compositions. Parameters including elevation, ice age, and concentrations of dust, chloride, sulfate, and sodium, significantly (p ≤ 0.05) correlated with microbial community compositions (Additional file 1: Table S4). This further supports above discussion that explains the potential differences between the microbial communities of the two ice cores, and is consistent with many previous reports that the microbial communities archived in glacier ice often reflect the differences in many physicochemical parameters such as dust concentration [10–12] and some ion concentrations [13, 14]. The significant correlations between microbial community compositions and environmental parameters of ice samples indicated that the ice core microbial communities may possibly reflect climate conditions at the time they were deposited. We note that other possibilities might also influence the microbial communities, such as the deposition-to-accumulation process as discussed above and the potential post-deposition microbial activity on glacier surfaces.

### Ice-archived viruses

We focused on the virus communities in two ice samples (D25 and D49) from the S3 ice core. The samples were selected based on their difference in ice age (~355 versus ~14,400 years old), climate conditions (colder versus warmer based on the δ^18^O data, not shown), and dust concentrations, which are up to 10 times higher in the D49 sample (Additional file 1: Table S3). Viruses were concentrated from 0.22-μm-pore-sized filtrate, which excluded intracellular viruses including temperate viruses [61], and then treated with DNase to remove free DNA. Counts of VLPs in the two samples were below the detection limit using a wet-mount method (<10^6^ VLPs ml^−1^ [62];). Thus, we applied the low-input quantitative viral metagenomic sequencing that was previously established to study seawater viral communities [46, 47, 63, 64], to the viral concentrates in our low-biomass glacier ice samples. After sequencing, quality control, and de novo assembly, we obtained 1849 contigs with a length of ≥10 kb (Additional file 1: Table S5). Overall, VirSorter predicted 43 “confident” viral contigs (≥10 kb in size and categories 1, 2, 4, or 5; Additional file 1: Table S5 [65]), which were grouped into 33 vOTUs (viral OTUs) using currently accepted cutoffs that approximate species-level taxonomy [35, 50, 66]. This is a small number of viral species compared to well-studied and relatively easy-to-process sample types (e.g., global ocean samples [35, 37, 66]), and may not represent the entirety of dsDNA viral diversity in the glacier ice environments. However, it is on par with recent reports in other more challenging systems such as soils where, for example, 1.4% of assembled contigs were predicted as “confident” viruses and 53 long (≥10 kb) viral genome fragments were recovered from eight viromes [67]. On average, 1.4% (2.2 and 0.6% for D25 and S3.49, respectively) of the quality-controlled reads were recruited to these vOTUs (Additional file 1: Table S5). Low percentage of reads recruited to predicted viral sequences is not unusual for low-input viromes, and consistent with previous studies from more diverse communities (e.g., as low as 0.98% [35, 67]).

While previous studies have detected tomato mosaic tobamovirus RNA and estimated VLP concentrations in ancient glacier ice [3, 27], this is the first report of viral genome fragments assembled de novo from such an environment. Rarefaction curves were constructed (see “Materials and methods”) and showed that both viromes approached saturation of long vOTUs (≥10 kb) at the sequencing depth used in this study (Additional file 2: Fig. S3), though we note that this analysis may underestimate the total viral diversity in these samples because (i) these rarefaction curves missed any potential virus whose genome was not extracted, sequenced, or assembled from the samples, and (ii) low-input libraries have to be PCR-amplified prior to sequencing (15 PCR cycles in this study), and this can underestimate the total diversity within a library due to PCR duplicates and skew the shape of rarefaction curves [68].

### Ice viral communities consist of mostly novel genera and differ between depths

**Fig. 4:**
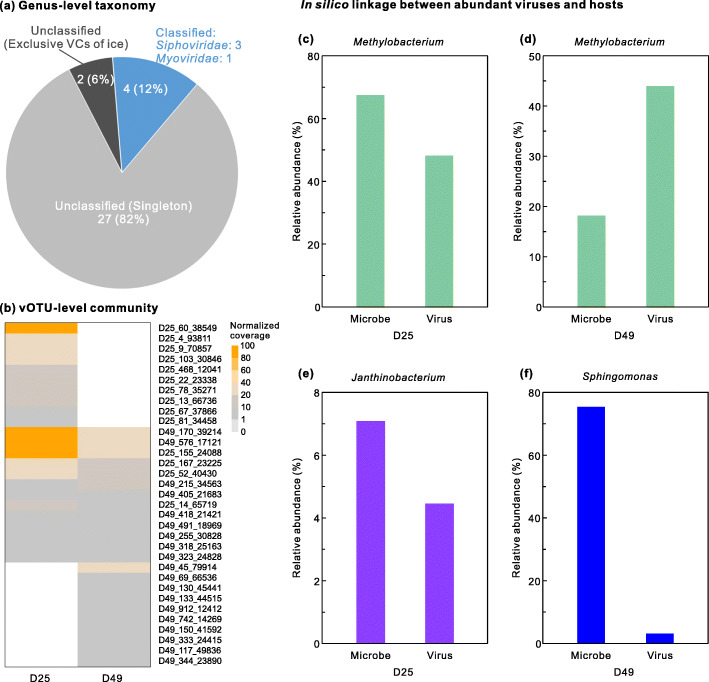
Taxonomies (**a**), communities (**b**), and host linkages (**c**–**f**) of 33 vOTUs recovered from two glacier ice samples. **a** Viral taxonomy was assigned by comparing genome-content-based network analysis of the 33 glacier vOTUs and 2304 known viral genomes in the NCBI RefSeq database using vConTACT v2 (see “Materials and methods”). vOTUs were classified into three groups: “Singletons” (gray) that had no close relatives; “Exclusive VCs” (black) that were viral clusters (VCs) of exclusively glacier ice vOTUs; and “Classified VCs” (blue) which included glacier ice vOTUs and Refseq viral genomes. **b** The normalized coverage of these 33 vOTUs was generated by mapping quality-controlled reads to vOTUs, and was normalized to per gigabase of metagenome. **c**–**f** Relative abundances of three abundant (>1.0%) microbial genera and their viruses: **c** *Methylobacterium* in D25, **d** *Methylobacterium* in D49, **e** *Janthinobacterium* in D25, and **f** *Sphingomonas* in D49. Relative abundances of microbes are based on 16S rRNA amplicon sequencing, and vOTUs are based on their coverages generated by mapping quality-controlled reads to vOTUs. Viruses were linked to hosts in silico by three methods: Blastn, VirHostMatcher, and CRISPR matches (see “Materials and methods”)

With 33 vOTUs (length ≥10 kb) obtained from the two S3 ice samples, we then evaluated how viruses in this unexplored extreme environment compared to known viruses. Because viruses lack a single, universally shared gene, taxonomies of new viruses are now commonly established using gene-sharing analysis from viral sequences [69]. In our dataset, that meant comparing shared gene sets from 33 vOTUs with genomes from 2304 known viruses in the NCBI RefSeq database (version 85; Additional file 1: Table S6) using vConTACT version 2 [69]. Such gene-sharing analyses produce viral clusters (VCs), which represent approximately genus-level taxonomic assignments [37, 69, 70]. Of the 33 vOTUs, four were clustered into four separate VCs containing RefSeq viral genomes, two formed a VC with only ice vOTUs, and the other 27 vOTUs remained isolated as singletons or outlier vOTUs (Fig. 4a; Additional file 1: Table S6). Therefore, only four vOTUs (12%) could be assigned a formal taxonomy: they belonged to four different genera in the families *Siphoviridae* (three genera) and *Myoviridae* (one genus) within the order *Caudovirales* (Additional file 1: Table S6). These taxonomic results indicate that glacier ice has a diversity of unique viruses, consistent with, but much higher than, other environmental studies in oceans (52% unique genera) [37] and soils (61% unique genera) [71].

We then explored the environmental distribution of these 33 glacier viruses by recruiting metagenomic reads from a range of environments including global ocean [66], Arctic sea ice and ancient permafrost brine (cryopeg) [42], soils [72, 73], lakes [74, 75], deserts [76–79], air [80, 81], cryoconite [40], and Greenland ice sheet [40] (225 metagenomes total). None of our 33 glacier vOTUs was detected in any of the tested metagenomes, indicating that the glacier ice archived unique viral communities compared to other environments, at least based on the viral populations recovered here. This may be due to the fact that the glacier viruses were “frozen” several thousands of years ago, that these ancient glacier viruses are unique from the viruses in the modern environments that have probably been evolving for a long time, or that these preserved glacier viruses were not transported from those regions where the tested metagenomes were sampled. Unfortunately, the lack of viromes from ancient glacier ice limits worldwide glacier habitat analyses. However, it is promising that the “black box” of the archived ancient virus in glacier ice can now be gradually opened as the technologies to generate and study clean and low-biomass viromics, including a modern viromic toolkit [36], are becoming available [46, 47, 63, 64].

Next, we looked more closely at the vOTU (~species) level to compare viral communities obtained from the archive of two depths of the S3 ice core. With standard read-mapping to 33 vOTUs (see “Materials and methods”), we found that the glacier ice from the two depths contained a mix of shared and depth-unique vOTUs (Fig. 4b; Additional file 1: Table S7). A mix of shared and depth-unique microbes was also observed for these samples (Fig. 3a; Additional file 1: Table S2). Previous studies have also reported different microbial community structures in ice samples collected from different depths of the same ice core, which probably reflects differences in the environmental conditions at the time the ice was deposited [11, 82]. Interestingly, three vOTUs were abundant (relative abundance >10%) among the recovered vOTUs in both depths: D49_170_39214, D49_576_17121, and D25_155_24088 (vOTU names, Fig. 4b; Additional file 1: Table S7). This suggests that these viruses may be active in these ice cores or that a large number of virus particles were initially deposited so that a sufficient amount was still intact for DNA extraction and sequencing after being frozen for potentially 15,000 years.

### Glacier ice viruses are predicted to infect dominant glacier ice microbes

Microbial analysis found that both the D25 and D49 samples were dominated by the bacterial genus *Methylobacterium*, an unclassified genus within the family *Methylobacteriaceae*, and genus *Sphingomonas*, with relative abundances of 18.2–67.5%, 5.0–8.3%, and 1.4–75.3%, respectively (Fig. 3a). In addition, the genera *Janthinobacterium* (7.1%) and *Herminiimonas* (6.6%) were also abundant in D25, but were absent or rare (<0.01%) in D49 (Fig. 3a). All of these genera are common abundant microbial groups in glaciers [4, 15, 17, 57–59]. In addition, many members belonging to these genera are psychrophilic bacteria and have been revived and isolated from glacier ice, such as *Sphingomonas glacialis* C16y, *Sphingomonas* sp. V1, *Methylobacterium* sp. V23, *Janthinobacterium svalbardensis* JA-1, and *Herminiimonas glaciei* UMB49 [18, 57, 83–85]. These results indicate that the ice serves as an archive for abundant taxa that are likely equipped with genomic adaptations to cold conditions and might revive and be introduced into ecosystems after the glaciers melt in the future.

We then explored the potential impacts of viruses on these abundant microbes by linking viruses to their hosts in silico. Hosts for the 33 vOTUs were predicted using three in silico methods: similarities in viral and bacterial nucleotide sequences [37, 86], composition [87], or CRISPR spacer matches [37]. The sequence similarity method (Blastn) predicted hosts for 14 of the 33 vOTUs (Additional file 1: Table S8), whereas the sequence composition method (VirHostMatcher) linked nine vOTUs to microbial hosts (Additional file 1: Table S9; see “Materials and methods”). The CRISPR method matched hosts for two vOTUs (Additional file 1: Table S10), one of which was also linked to the same host at genus level by the sequence similarity method but none of them was matched by the sequence composition method (Additional file 1: Tables S7, S8 & S9). Although only about half (18 of 33 vOTUs) of the vOTUs were linked to a host by at least one of the three methods, these host predictions indicated that viruses in glacier ice were infectious to microbes at some time (whether before and/or after ice formation) in these extreme cold and high-elevation environments, and that they probably played an important role in modulating microbial communities.

The predicted host genera that were most abundant in the same ice cores included *Methylobacterium*, *Sphingomonas*, and *Janthinobacterium* (Fig. 3a; Additional file 1: Table S2). Many members of these genera are psychrophilic bacteria as mentioned above. The relative abundance of *Methylobacterium*-associated vOTUs was high in both D25 (67.5%) and D49 (18.2%), which was consistent with the dominance (48.2% and 44.0%, respectively) of this bacterial genus in the microbial communities of both samples (Fig. 4c, d). Similarly, *Janthinobacterium*-linked viruses were detected with a high relative abundance of 7.1% in the D25 sample, where microbial community was found to be dominated by the genus *Janthinobacterium* with 4.5% relative abundance (Fig. 4e); *Sphingomonas*-associated viruses represented 3.1% of communities in the D49 sample, while members of *Sphingomonas* accounted for 75.3% of the microbial profiles in this sample (Fig. 4f). The relatively high abundance of these genera and their associated viruses suggests that the recovered viruses infected abundant microbial groups and thus might play a major role in this extreme ecosystem by influencing their hosts when they are active, although it is still uncertain when the infections occurred. Notably, no host could be predicted for about half of the vOTUs, partly due to the limitations of available reference databases and techniques used for host prediction [86]. As methods improve and host databases expand (e.g., Genome Taxonomy Database [88] and metagenome-assembled genomes from glacier ice), continued studies will likely provide more complete understanding of the relationship between viruses and their microbial hosts in the ice cores.

### Temperate viruses likely dominate glacier ice environment

Having investigated virus-host pairs, we then explored the lifestyle (i.e., temperate or virulent) of the 33 vOTUs we were able to recover here. Interestingly, 14 (42.4%) vOTUs were identified as putative temperate viruses (see “Materials and methods”; Additional file 1: Table S11). Though a small dataset, the percentage of identifiably temperate phages in glacier ice was 3.2-, 8.4-, and 14.1-fold more than that in gut (13% [89]), soil (5% [67, 71]), and marine (3% [66]) viruses, respectively, detected by the same method. Several specificities of glacier ice habitats may explain such high percentage of temperate phages. Glacier ice is an extreme habitat for microbes and viruses with low temperature, high UV, and low nutrient concentration, in which microbes are usually under poor growth conditions, and microbial density is very low (i.e., 10^2^–10^4^ cells ml^−1^ [4]) compared to most other environments (e.g., seawater contains 10^4^–10^6^ cells ml^−1^ [7]). Previous reports highlighted how the frequency of temperate viruses is influenced by environmental conditions (reviewed in [39, 90]) and that temperate viruses tend to be more abundant compared to virulent viruses under extreme environments of low temperature [91, 92], high latitude [93], low nutrients [94], and low host concentrations [95]. We hypothesize that, as similar to other extreme and low-nutrient environments, temperate phages are selected for and favored before being frozen in glacier ice. Mechanistically, this selection process likely happened on the glacier ice surface, as microbes on the surface snow of the glacier are exposed to nutrients, light, and possible melt water when temperature is high in the summer, and they may still be active and undergo a selection progress on glacier surfaces (reviewed in [9]). This progress may lead to substantial size fluctuation of microbial populations and bottleneck events, which have been shown to favor temperate viruses [90, 96]. Overall, our data suggest that temperate phages likely dominate glacier ice environment and highlighted the importance to specifically target these viruses (e.g., intracellular viruses) in future studies of viruses archived in glacier ice.

### Insights into the gene content and genome organization of viruses infecting *Methylobacterium*

Microbial analyses and viral host predictions found that both microbial members within the genus *Methylobacterium* and their associated viruses were abundant in the two studied glacier ice samples. Members of the genus *Methylobacterium* were reported to dominate the microbial community in ancient ice cores from many previous studies (e.g., [4, 12, 16, 25]) including several microbial investigations of the Guliya ice cap ice cores using culture-dependent methods about two decades ago [18, 23, 57], and they are widely distributed in natural environments. For example, the genus *Methylobacterium* contains 47 validly published isolates at the time of writing (https://www.bacterio.net/genus/methylobacterium) from environments including air, aquatic sediments, fermented products, freshwater, plants, and soil (summarized in [97]). The broad distribution indicates their ability to live in a wide range of environments. The viruses infecting *Methylobacterium* may also have significant ecological roles, so next we evaluated the environmental distribution of viruses infecting *Methylobacterium* and the genome features of *Methylobacterium*-linked glacier viruses and their closely related viruses from other environments.

*Methylobacterium*-associated viruses were obtained from environmental viromes including global oceans [35], Arctic sea ice and ancient permafrost brine (cryopeg) [42], soils [72, 73], lakes [74, 75], deserts [76–79], air [80, 81], cryoconite [40], and Greenland ice sheet [40], by the same method as for glacier-ice viruses. In addition, prophages were extracted from 131 *Methylobacterium* genomes from the RefSeq database (release v99). Only six *Methylobacterium* viruses were obtained from the environmental metagenomes, including three from global oceans [35], two from lake water [75], and one from a desert salt pan [77], while 478 prophages were detected from 127 out of 131 *Methylobacterium* genomes that were from diverse environments such as plant, soil, freshwater lake, drinking water, ocean water, salt lake, air, and ice (Additional file 1: Table S12).

**Fig. 5:**
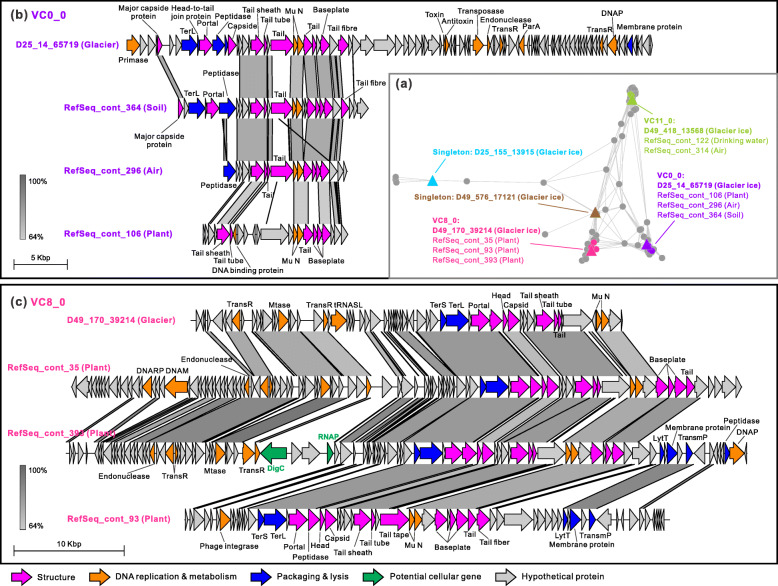
Genome content–based network (**a**) and genome organization (**b**–**c**) of viruses infecting *Methylobacterium*. **a** A gene content–based network was built to evaluate the relationship of five glacier-ice viruses to 484 viruses from other environments, all predicted to infect *Methylobacterium* (see “Materials and methods”). For clarity, viruses that were not connected to any of the five glacier-ice viruses were excluded from the network. Each node represents a virus, with glacier-ice viruses and others shaped in triangle and circle, respectively. The edge between nodes indicates the distances between two viruses. Viral clusters (VCs) are generated by vConTACT v2, and viruses that belonged to the same VC are indicated in the same color. In each VC, the name and source environment of each member are indicated, with glacier-ice virus at the top. All gray nodes represent viruses from other environments that did not share VC with any glacier-ice virus. **b**–**c** Genomic organization and comparison of *Methylobacterium* viruses that are longer than 15kb in VC0_0 and VC8_0 from (**a**). Only glacier viruses and their closely related viruses with genome size more than 15kb were illustrated, including four and four viruses from VC0_0 (**b**) and VC8_0 (**c**), respectively. Viral contigs were compared in terms of gene similarity, order, and direction (i.e., leftward or rightward arrow). Genes are coded in color based on their putative biological function. Potential microbial genes were identified by CheckV (see “Materials and methods”) and marked in green color. The predicted protein with no functional annotation is classified as “Hypothetical protein” and colored in gray. The gray lines indicate the amino acid identities between genes, as illustrated in the scale bar. Abbreviations: TransR, transcriptional regulator; MTase, mRNA methyltransferase; tRNASL, tRNA-splicing ligase; terS, terminase small subunit; terL, terminase large subunit; Mu N, Mu *N* gene product; DNARP, DNA repair protein; DNAM, DNA methylase; DNAP, DNA polymerase; RNAP, RNA polymerase; LytT, lytic transglycosylases; TransmP, transmembrane protein; DigC, diguanylate cyclase

A genome content–based network was built to evaluate the relationship of five glacier-ice viruses with 484 viruses from other environments, all predicted to infect *Methylobacterium* (Fig. 5a). In the network, two glacier virus (D25_155_13915 and D49_576_17121) were separate from any other viruses (i.e., they were singletons), the other three glacier viruses formed three VCs with eight prophages (i.e., VC0_0, VC8_0, and VC11_0; assessed with confidence scores by vConTACT v2 [69]). The vOTU D49_418_13568 was associated with viruses from air and drinking water (VC11_0), vOTU D49_170_39214 (VC8_0) was clustered with viruses from plants, while D25_14_65719 (VC0_0) was clustered with plant, air, and soil viruses (Fig. 5a and Additional file 1: Table S12). Notably, most of the associated prophages within the three VCs were from plant, soil, or air, which might be the habitats from which the glacier *Methylobacterium* hosts and viruses originated.

We next evaluated the genome content and organization of above-clustered *Methylobacterium* viruses using two glacier-ice viruses and six prophages that were longer than 15 kb (Fig. 5b, c). The glacier viruses shared a similar genomic content and arrangement with the prophages in the same VC, especially for the phage structure genes including the portal, capsid, tail, and baseplate genes (Fig. 5b, c). Notably, all these viruses contained two copies of Mu *N* genes that were located near the tail and baseplate genes (Fig. 5b, c and Additional file 1: Table S13). The *N* gene product (i.e., DNA circularization protein) has been reported as a multifunctional protein that is injected into the host cell along with the infecting phage DNA and is involved in tail assembly, as well as the protection and circularization of the infecting DNA [98–100]. Phylogenetic analysis of the 16 (two copies in each of eight viruses) *N* genes showed that these genes formed two clusters, and each cluster included one of the two copies of *N* genes from all eight *Methylobacterium* viruses (Additional file 2: Fig. S4). These results indicated that the two copies of *N* genes likely evolved independently in the same virus, though this is still unclear with the limited information presented in this study. In agreement with the genome-based network analysis, the viruses from the same VC clustered together based on either copies of the *N* genes (Fig. 5a; Additional file 2: Fig. S4), indicating strong conservation of *N* genes in the *Methylobacterium* viruses.

Taken together, the viruses infecting *Methylobacterium* appear to be abundant in the glacier ice and are related to viruses infecting *Methylobacterium* strains in plant and soil habitats. This is consistent with a previous report that the main source of dust deposited on Guliya ice cap likely originates from the soils [101]. This points to a potential long-standing association between phages and their host in the *Methylobacterium* genus, possibly over more than tens of thousands of years, and highlights how some bacteria and phages can seemingly stably coexist in the environment, as argued in other studies (e.g., [102, 103]).

### Glacier ice viruses unravel novel auxiliary metabolic genes (AMGs) potentially influencing host chemotaxis

Virus-encoded auxiliary metabolic genes (AMGs) are microbial-derived genes that can modulate host metabolism during infection and have been reported in viruses from diverse ecosystems such as marine water [37], soil [67, 71], animal host (e.g., rumen [104]), and some extreme environments (e.g., Arctic cryopeg brine and sea ice [42]). Here, we begin to explore the AMGs of viruses archived in glacier ice. Briefly, 1466 predicted genes from the 33 vOTUs (length ≥ 10 kb) were queried against functional databases by DRAM-v (see “Materials and methods”), which resulted in about half genes (n = 779) matching annotated sequences in KEGG or PFAM databases (Additional file 1: Table S13). These annotations will potentially enable the datasets as valuable public resource of ancient viral genes.

Four putative AMGs were identified from these annotated genes (Additional file 1: Table S14). Two of them were previously reported, including concanavalin A-like lectin/glucanases superfamily and sulfotransferase [37, 71]. The former one was associated with virus-encoded glycoside hydrolase that was potentially involved in pectin cleavage, thus, further potentially facilitating microbial carbon degradation and utilization through cleaving polymers into monomers and influencing the carbon cycling [71]. The later one was associated with sulfation that contributes to the transfer reaction of the sulfate group from the donor (e.g., 3′-phosphoadenosine 5′-phosphosulfate) to an acceptor that can be a number of substrates, and can potentially play a key role in biological processes such as cell communication and growth [105]. The other two AMGs, *motA* and *motB*, that were potentially relevant to cell flagella assembly (Additional file 1: Table S14), were never reported previously as AMGs in viral contigs, though our screening of 848,507 viral contigs in the Global Ocean Viromes 2.0 dataset (GOV 2.0 [66]) identified *motA* or *motB* genes from 70 high-quality viral contigs in 52 viromes including 23, 15, and 14 viromes from surface water, mesopelagic water layers, and deep chlorophyll maximum layers, respectively (Additional file 1: Table S15), indicating their broad distribution in the ocean environment. These AMGs can potentially offer new insights into how viruses manipulate microbial metabolisms when they might have been active ~14,400 years ago before being frozen. Here, we further focused on the two novel AMGs and discussed how they potentially influence the metabolisms of microbial hosts in glacier ice. These two novel genes were motility genes (*motA* and *motB*) from the same vOTU D25_22_20338 (Additional file 1: Table S14; Additional file 2: Fig. S5a).

Fueled by ion flow, bacterial flagella are turned by rotary motors which consist of the stator and the rotor [106]. Analyses of AMGs in glacier-ice viruses revealed that the vOTU D25_22_20338 encoded two membrane-embedded proteins, MotA and MotB (Additional file 2: Fig. S5a), which compose the stator of a flagellar motor. In bacteria, MotA/MotB protein complexes function in delivering protons to the rotor, thus generating a proton motive force as the energy source to rotate the rotor [107]. Chemotaxis plays a central role in controlling the rotational direction of flagellar motors, which allows bacteria to respond to environmental stimuli [108]. Considering the harsh environment associated with nutrient deficiency in glacier ice [109], we speculate that viruses potentially hijacked these motility genes (i.e., *motA* and *motB*) to facilitate nutrient acquisition of their hosts.

We then explored the functionality and evolution of the two novel AMGs (i.e., *motA* and *motB*). The protein sequences of the two novel AMGs were structurally modeled using Phyre2 [110], and the results showed that both had 100% confidence scores that linked them to their closest template protein (Additional file 2: Supplementary Fig. S5bc). MotB uses a conserved peptidoglycan-binding motif to anchor the stator complex to the peptidoglycan layer around the rotor [111], and this motif was identified in the virus-encoded MotB (Additional file 2: Fig. S5e). Though MotA lacks a conserved motif (Additional file 2: Fig. S5d), it functions as a complex and is co-transcribed and translated with MotB [112]. Together, these in silico analyses suggested that these AMGs are likely functional. Evolutionarily, both AMGs were deeply isolated from all clades with their mostly close microbial homologs (Additional file 2: Supplementary Fig. S6ab). These phylogenetic results limited us to further identify potential horizontal gene transfer events of these AMGs from hosts to viruses, while they suggested that these genes found in the ancient glacier-ice viruses recovered in this study are very distinct from known microbial sequences in modern environments.

In summary, these findings about AMGs can potentially provide a glimpse into how glacier-ice viruses, in the Guliya ice cap, manipulate host metabolism and hence likely affect biogeochemical cycles when they were active before being frozen. We note that all these speculations are based on in silico analyses; future experiments are necessary to validate the activity and function of these potential virus-encoded proteins.

Many studies have demonstrated microbial activity on the glacier surfaces especially in the cryoconite holes in summer (e.g., [113, 114]), including glaciers from Tibetan plateau region [9, 82]. However, the surface activity may vary from glaciers with different location, elevation, radiation, and surface temperature. Guliya ice cap is located at middle latitude (35.25°N; 81.48°E) of Tibetan Plateau, and the summit elevation is about 6710 m above sea level. The surface temperature of the summit is below the water frozen point (0°C) during most of the time in a year around, while in the summer, the Guliya surface temperature could approach near or above 0°C for short periods and has strong sunlight input; this likely leads to produce some melt water on the glacier surface, which was supported by the observation of melt layers (i.e., clean and transparent ice) in the ice core (data not shown). Therefore, there is likely microbial activity on the surface of Guliya ice cap before microbes were “permanently” frozen. In addition to the glacier surface, some studies have hinted at the possibility of microbial activity in frozen glacier ice based on the detection of some excess gases (e.g., CO_2_, CH_4_, and N_2_O) at some depths, which may be produced by post-depositional microbial metabolism [24–26]. However, without direct observational measurements, it remains controversial in whether there is in situ microbial activity in glacier ice after being frozen. We anticipate future studies could better articulate the potential microbial activity in glacier environments including the surface and englacial ice (i.e., after being frozen). Here, we propose next-step experiments trying to explore the “activity” questions described above. Ideally in the field work, we could sample the time-series snow before deposition (i.e., from air) and after deposition (i.e., from different depths of glacier ice surface) and compare the microbial communities of matched snow samples from before and after deposition. The results from comparison will help us understand if there is activity and how communities change on the glacier surfaces. In addition, some specific microbial groups (e.g., Cyanobacteria and Chloflexia) may be used as indicator of surface growth, as they need light to grow and may “bloom” on the glacier surface [82]. In the lab, microbial activity in glacier ice could be measured using the BONCAT-FACS method [115] through comparing the potential change of microbial communities of the sample replicates after incubations under various conditions in temperatures (< 0°C) and times.

## Conclusions

Glaciers potentially archive environmental conditions and microbes over tens to hundreds of thousands of years. Unfortunately, glaciers around the world, including those from Tibetan Plateau and Himalaya, are rapidly shrinking, primarily due to the anthropogenic-enhanced warming of Earth’s ocean-atmosphere system [116]. Such melting will not only lead to the loss of those ancient, archived microbes and viruses but also release them to the environments in the future. To begin accessing these archived microbes and viruses, we expanded upon prior in silico [17] and experimental decontamination methods to remove microbial contaminants from ice core surfaces [51–54] and optimized similar preparation methods for viruses. Application of these new ultra-clean methods to ~14,400-year-old glacier ice presents the first glimpse of past microbial and viral communities archived in glacier ice from the Tibetan Plateau. These efforts revealed microbiological findings concordant with other ice cores and provided a first window into viral genomes, communities and their ecology, functions, and origin in ancient glacier ice in this remote part of the world.

Future work will benefit from emerging technologies to detect microbial growth (e.g., BONCAT-FACS [115]), better capture of very small diverse vOTUs and niche-defining hypervariable regions (VirION [117]) including ssDNA [118] and RNA viruses [119, 120], and high-throughput cultivation (e.g., Microfluidic Streak Plates method [121]). Earth is now squarely in the Anthropocene, and human activities are impacting the planet and its interconnected ecosystems in ways no single species has done before [122]. Fortunately, application of advanced research capabilities for the intensive study of ice-core-derived biotic and abiotic information may reveal the primary drivers of both natural (pre-anthropogenic) and anthropogenic variations in microbial evolution.

## Materials and methods

### Sterile artificial ice core sections and mock “contaminants”

An artificial ice core was constructed from sterile water, which was pre-filtered through a Millipore system (Cat No. MPGP04001, MillipakR Express 40 Filter, Merck KGaA) outfitted with a 0.22-μm mesh final filter and autoclaved at 121°C for 30 min, then frozen at −34°C for 12–24 h in a 2-L sterile plastic cylinder (Nalgene). The cylinder was transferred from −34 to −5°C and kept at that temperature overnight to reduce the possibility of fracturing (which is caused by sudden temperature changes) before placing it at room temperature for about 30 min to melt the surface ice and expose the underlying ice core.

*Cellulophaga baltica* strain #18 (CBA 18; NCBI accession No. CP009976) was cultured in MLB medium (15 g sea salts (Cat No. S9883, Sigma), 0.5 g bacto peptone, 0.5 g yeast extract, 0.5 g casamino acids, 3 ml glycerol, and 1000 ml water) stationary overnight at room temperature. The cell concentration was measured by epifluorescence microscopy after the cells were captured on a 0.22-μm-pore-sized filter (Cat No. GTTP02500, Isopore) and stained by SYBR Green (Cat No. S9430, Sigma) as described previously [123] with some modifications. Briefly, cells on the filter were covered with several drops of 20×SYBR Green (Cat No. S11494, Life Technologies). After 15 min of staining in the dark, the SYBR Green was carefully removed with a 50-μl pipette and by touching the backside of the membrane with a Kimwipe (Kimtech). The filter was mounted on a glass slide with freshly made anti-fade solution (1 mg ascorbic acid: 100 μl PBS: 100 μl glycerol) and a 25-mm^2^ cover slip. Cells on the filter were counted using epifluorescence microscopy (Zeiss Axio Imager.D2) with >350 cells or >20 fields counted, which was a reliable threshold to estimate the total bacterial abundance [124].

*Pseudoalteromonas* phages strain PSA-HP1 (NCBI: txid134839) were harvested from 95% lysed plaque assays (agar overlay technique). The concentration of PSA-HP1 was counted by a wet-mount method using SYBR Gold (Cat No. S11494, Life Technologies) staining and glass beads as described previously [62]. The lambda phage DNA (100 μg/ml; 1.88×10^9^ copies/μl; genome size 4.8 kb) was purchased from Life Technologies (Cat. No. P7589). Above components (i.e., CBA 18, PSA-HP1, and lambda phage DNA) were combined in 1 ml ddH_2_O, which contained 1.00×10^6^ cells, 4.48×10^7^ viruses, and 1.88×10^8^ copies of lambda DNA to make the mock contaminants. The concentration of contaminant cells is approximate to the cell numbers in glacier ice (~10^2^–10^4^ cells/ml [4]) and a previous report of core exteriors (~10^2^–10^5^ cells/ml [52]). The 1 ml mixtures were spread evenly on the artificial ice core surface with sterile gloved hands. The ice core was cut into three equal-sized sections with a sterilized band saw, which was previously wiped with 75% ethanol and exposed to UV light for >12 h.

### Surface decontamination procedures

The decontamination procedure consisted of three steps (Fig. 1a) following a previously published method [52] with slight modifications. First, the exterior (~0.5 cm of the core radius) of the ice core was scraped away using a sterile band saw; second, the ice core was rinsed with 95% ethanol (v/v; Cat No. 04355223, Decon Labs) to remove another ~0.5 cm of the surface; third, a final ~0.5 cm of the surface was washed away with sterile water (Fig. 1a; Additional file 2: Fig. S1). After about 1.5 cm of the core surface was removed, the inner ice was the “clean” sample and collected for further analyses.

Two artificial ice core sections (sections 1 and 2) were processed using the decontamination procedure described above (Fig. 1a). The ice removed by the saw scraping (first step), water washing (third step), and the inner ice were collected as three different samples in sterile beakers. As a positive control, another ice core section was placed in a sterile beaker, which was not decontaminated (Fig. 1a). All sampling steps were conducted in a cold room (−5°C), which was exposed to UV light for more than 12 h before ice core processing to kill microbes and viruses in the air and on the surface of the instruments (e.g., band saw, washing systems, and flow hood; Additional file 2: Fig. S1). In addition, we performed the washings with 95% ethanol and water in the BioGard laminar flow hood (Baker Company, model B6000-1) to avoid environmental contamination (Additional file 2: Fig. S1). Ice samples were melted at room temperature. One milliliter of each melted sample was preserved at 4°C and used for nested PCR to detect the coated lambda DNA. Other volumes of each sample were subjected to concentrating the microbes and viruses using 100 kDa Amicon Ultra Concentrators (EMD Millipore, Darmstadt, Germany). Each sample was concentrated to 0.8 ml and then was used for DNA extraction.

### Guliya ice core sampling and physiochemical conditions

The plateau shallow core (PS core 34.5-m depth; 35°14′ N; 81°28′ E; 6200 m asl) and the summit core 3 (S3 51.86-m depth to bedrock; 35°17′ N; 81°29′ E; ~6710 m asl) were drilled on the Guliya ice cap in 1992 and 2015, respectively (Fig. 2a, b, c). Both cores were 10 cm in diameter, and the bedrock temperature at the S3 site was about −15°C [125]. Ice core sections (~1 m each) were sealed in plastic tubing, placed in cardboard tubes covered with aluminum, and transferred at −20°C by truck from the drill sites to freezers in Lhasa, by airplane to freezers in Beijing, by airplane to Chicago, and then by freezer truck to the Byrd Polar and Climate Research Center at The Ohio State University where they have been stored at −34°C. Five samples were collected from the PS core at depths of 13.34–13.50 (sample name D13.3), 13.50–13.67 (D13.5), 24.12–24.54 (D24.1), 33.37–33.52 (D33.3), and 34.31–34.45 (D34.3) m (Fig. 2c; Additional file 1: Table S3). These ice samples were decontaminated using the surface-decontamination procedure described above, and the inner ice was collected for further analysis. In addition, the ice removed from the saw scraping and water washing was also collected for two samples (D13.3 and D13.5) as described for the artificial ice core sections in order to evaluate the surface decontamination procedures using authentic ice samples. The microbial communities from two of the S3 core samples (D41 and D49) were published previously [17]. Another sample D25 (25.23–25.79-m depth; not published) was collected at the same time as the two samples mentioned above and was included in this study (Fig. 2).

Four controls were used to trace possible sources of background contamination during ice sample processing as described previously [17]. First, we assessed what microbes inhabited the air of the cold room laboratory in which the sampling took place. Cells from about 28 m^3^ of air were collected over 4 days of continuous sampling in the room using an air sampler (SKC Inc.) as described previously [17], during which the ice samples were processed at the same time. This provided an evaluation of the background contamination due to ice exposure to air during the processing (Sample AirColdRoom). Second, an artificial ice core was made from sterile water (as described above), which was frozen at −34°C for 12–24 h. This sterile core was processed in parallel with the authentic ice core samples through the entire analysis. This control allowed evaluation of contamination from the instruments used to process the ice (Sample ArtificialIce). Third, a blank control was established by extracting DNA directly from 300 ml of sterile water. This control allowed evaluation of contamination downstream of the ice processing, including the molecular procedures (DNA extraction, PCR, library preparation, and sequencing; Sample Blank). Finally, 30 μl of filtered and autoclaved water was subjected to standard 16S rRNA gene amplicon sequencing to check contamination from the sequencing procedures (Sample BlankSequencing).

A total of 300 ml of artificial ice, 300 ml of the blank control, and 100–300 ml each of the glacier ice samples were filtered through sterilized polycarbonate 0.22-μm-pore-sized filters (Cat No. GTTP02500, Isopore) to collect microbes including all bacterial/archaeal cells larger than 0.22 μm. The filters were preserved at −20°C until DNA extraction (within 24 h). Viruses in the filtrate of two samples (D25 and D49) were concentrated to 0.8 ml using 100 kDa Amicon Ultra Concentrators (EMD Millipore, Darmstadt, Germany) and preserved at 4°C until DNA extraction (within 24 h). To check for possible cross contamination among samples and potential viral contaminants introduced to the samples during processing, 1 ml of 0.22-μm-pore-size filtrate from the water of the Olentangy River (named RiverV; 39°59′52″ N, 83°1′24″ W, Columbus, Ohio) was co-processed in parallel with samples D25 and D49 throughout the entire analyses. All the biological work in this study after the ice sampling in the cold room laboratory was performed in a hood within a small (~2 m^2^ in area) room that is reserved for microbial experiments with low-biomass samples. The hood was exposed with UV light for more than 1 h before experiments.

Concentrations of insoluble dust, major ions, and oxygen isotopes of glacier ice were analyzed as described previously [126]. The development of the chronologies for the two ice cores from which the samples were collected is discussed in Additional file 1: Table S3, where the ages of the samples were provided.

### Genomic DNA extraction

The viral concentrates from samples D25, D49, and RiverV were subjected to isolating genomic DNA as previously described [45]. Briefly, viral concentrates were treated with DNase (100 U/ml) to eliminate free DNA, followed by the addition of 100 mM EDTA/100 mM EGTA to halt DNase activity; genomic DNA was then extracted using Wizard® PCR Preps DNA Purification Resin and Minicolumns (Cat. No. A7181 and A7211, respectively; Promega, USA) [45]. Viral abundance, calculated prior to DNA extraction, was obtained by enumerating and comparing the counts of VLPs and beads (with a known concentration) using the wet-mount method [62].

Genomic DNA from all other samples was isolated with a DNeasy Blood & Tissue Kit (Cat No. 69506, QIAGEN) according to the manufacturer’s instructions, with an additional step of beating with beads to disrupt bacterial spores and Gram-positive cells before cell lysis by homogenizing at 3400 RPM for 1 min with 100 mg of autoclaved (121°C for 30 min) 0.1-mm-diameter glass beads (Cat No. 13118-400, QIAGEN) in a MiniBeadBeater-16 (Model 607, BioSpec Products).

### Nested PCR

Nested PCR experiments [127] were performed during the clean surface decontamination procedures, using two pairs of primers designed to detect lambda phage DNA in the artificial ice section samples. The external primer set LamouterF (5′-CAACTACACGGCTCACCTGT-3′) and LamouterR (5′-ACGGAACGAGATTTCCGCTT-3′) amplifies a 674 bp fragment, and the nested primer set LaminnerF (5′-GAAGCTGCATGTGCTGGAAG-3′) and LaminnerR (5′-CACACTCTGGAGAGCACCAC-3′) amplifies a 189 bp fragment within the previous fragment. In the first PCR with the external primer sets, the 25 μl reaction mixture consisted of 12.5 μl 2× commercial mix (Cat No. M712B, GoTaq® Green Master Mix, Promega), 1.25 μl of each external primer (LamouterF/LamouterR, 10 uM), 5.0 μl template DNA, and 5 μl of ddH_2_O. The amplification included a 5-min denaturation step at 95°C, followed by 40 cycles of 30 s at 95°C, 30 s at 56°C, and 50 s at 72°C, with a final extension of 5 min at 72°C. For the nested PCR, the reaction mixture was identical to the first PCR, except that 5.0 μl of the first PCR product and 1.25 μl of each nested primer (LaminnerF/LaminnerR, 10 μΜ) were included. The amplification conditions were also identical to the first PCR except for the extension time of 20 s at 72°C for 40 cycles of amplifications.

For each of artificial ice section samples (i.e., Cut1, Wash1, Inner1, Cut2, Wash2, Inner2, and Mix; Fig. 1a), 5 μl of melt water served as the DNA template in the first PCR. In addition, nested PCRs were performed using diluted lambda DNA (1.88×10^4^, 10^3^, 10^2^, 10^1^, 10^0^ and 10^−1^ copies, respectively) as templates to serve as a reference. A negative control was conducted using 5 μl of ddH_2_O as template.

### Real-time quantitative polymerase chain reaction (qPCR)

Each 20 μl reaction for qPCRs contained 10 μl of 2× QuantiTect SYBR Green PCR Master Mix (Cat No. 204143, QIAGEN), 0.5 μl of each primer (10 μM), 3 μl of template DNA, and 6 μl of RNase-free water. All reactions were performed in triplicate, using an Illumina Eco cycler (Cat No. 1010180).

Total bacterial and archaeal biomasses of the glacier ice samples and the “background” controls were estimated using qPCR after isolating DNA. The primer set 1406f (5′-GYACWCACCGCCCGT-3′) and 1525r (5′-AAGGAGGTGWTCCARCC-3′) was used to amplify bacterial and archaeal 16S rRNA genes [128]. Thermocycling consisted of an initial polymerase activation and template DNA denaturation step at 95°C for 15 min, followed by 40 cycles of 95°C for 15 s, 55°C for 30 s, and 72°C for 15 s. A standard curve was generated with a PCR product using primers 1406f/1525r from CBA 18 (NCBI accession number of the complete genome, CP009976).

Total numbers of CBA 18 in each of the artificial ice samples (i.e., Cut1, Wash1, Inner1, Cut2, Wash2, Inner2, and Mix; Fig. 1a) were quantified using the primer set Cbal18M666_05390F (5′-ACGTACAAATAAGGAGAATGGCTT-3′) and Cbal18M666_05390R (5′-AGCGCTAATCCCTGTTGAGA-3′), which specifically targets a 61 bp fragment of an ATP synthase subunit C of CBA 18, with thermocycling: 95°C for 15 min, 45 cycles of 95°C for 15 s, 60°C for 30 s, and 70°C for 25 s. Similarly, total PSA-HP1 numbers of these samples were quantified using strain-designed primer set 10-94a_dF (5′-TCTCTCGTCTTAATGACTTTCATCAT-3′) and 10-94a_dR (5′-TTCTTTCTCAACTTCCTGCTCTAA-3′), with the identical thermocycling conditions, except that 50 cycles of amplifications were conducted. The standard curves of the above two qPCRs were generated with the PCR products from their primer sets and strains, respectively.

### Tag-encoded amplicon sequencing of the microbial community

Bar-coded primers 515f/806r [129] were used to amplify the V4 hypervariable regions of 16S rRNA genes of bacteria and archaea for all the glacier ice samples and the “background” controls. Resulting amplicons were sequenced by the Illumina MiSeq platform (paired-end reads) as described previously [129]. These experiments were performed at Argonne National Laboratory.

### Amplicon sequence analysis

Sequences with an expected error >1.0 or length <245 nt were excluded from the analyses [130]. The remaining sequences were truncated to a constant length (245 nt). Various analyses were conducted using the QIIME (Quantitative Insights Into Microbial Ecology, version 1.9.1) software package [131] using default parameters, except that chimera filtering, operational taxonomic unit (OTU) clustering, and singleton exclusion were performed with QIIME through the UPARSE pipeline [130]. A phylogenetic tree was constructed with a set of sequence representatives of the OTUs using the method of FastTree [132]. Chimeras were identified and filtered by UPARSE with the UCHIME algorithm using the ChimeraSlayer reference database [133], which is considered to be sensitive and quick [134]. Reads were clustered into OTUs at 97% sequence similarity by UPARSE. A representative sequence from each OTU was selected for taxonomic annotation using the Ribosomal Database Project (RDP) classifier [135] from the RDP Release 11.5 database. Taxonomic assignments with <80% confidence were marked as unclassified taxa. Mitochondrial and chloroplast sequences were excluded from further analyses. A new profile of OTU composition for the ice samples was generated after in silico and proportional decontamination using R-OTU values >0.01 according to the method established previously [17]. Briefly, an R-OTU value was designated as the ratio between the mean “absolute” abundance of OTUs in “background” controls and ice samples; then, an approximated estimation of the “absolute” abundance of OTUs was calculated by multiplying the relative abundance of each OTU by the 16S rRNA gene copy number in a given sample (determined by qPCR). The OTUs with R-OTU values >0.01 were considered to be contaminants and were removed from the ice samples.

Each library was subsampled to the same sequencing depth before following analyses. Relative abundance of the microbial profiles was generated at genus and class levels. Principal Coordinates Analysis (PCoA) using weighted UniFrac metrics was performed to distinguish general distribution patterns of microbial profiles among all samples. The Mantel tests were conducted to evaluate the linkage between the microbial community structure and environmental parameters. The significance of the difference in microbial community between grouped samples (PS versus S3 core samples) was evaluated by analysis of similarity statistics (ANOSIM, number of permutations = 999), which was performed using functions in the Vegan package version 2.4-4 in R version 3.4.2 [136].

### Metagenomic sequencing of viral metagenomic dsDNA

The viral genomic DNA from three samples (D25, D49, and RiverV) was subjected to low-input library preparation pipeline using the Nextera® XT Library Prep Kit (Cat No. 15032354, Illumina) in the clean room, according to our methods described previously [46, 47, 63]. The metagenomes were sequenced by Illumina HiSeq 2000 platform (1×100 bp) at JP Sulzberger Genome Center at Columbia University.

### Viromic analysis and characterization of viral communities

All metagenomic analyses were supported by the Ohio Supercomputer Center. Viromic sequence data was processed using iVirus pipeline with default parameters described previously [35, 137]. Briefly, raw reads of three viromes, including two glacier ice samples (D25 and D49) and the River water control (RiverV), were filtered for quality using Trimmomatic v0.36 [138], followed by the assembly using metaSPAdes v3.11.1 (k-mer values include 21, 33, and 55) [139], and the prediction of viral contigs using VirSorter v1.0.3 in virome decontamination mode on CyVerse [65]. The viral contigs (categories 1, 2, 4, and 5) were first checked for contaminants by comparing them to viral genomes considered as putative laboratory contaminants (e.g., phages cultivated in our lab including *Synechococcus* phages, *Cellulophaga* phages, and *Pseudoalteromonas* phages) using Blastn. Then, they were clustered into vOTUs if viral contigs shared ≥95% nucleotide identity across 80% of their lengths as described previously [35, 49]. The longest contig within each vOTU was selected as the seed sequence to represent that vOTU. A coverage table of each vOTU was generated using iVirus BowtieBatch and Read2RefMapper tools by mapping quality-controlled reads to vOTUs, and the resulting coverage depths were normalized by library size to “coverage per gigabase of virome” [137]. Rarefaction curves of the two glacier ice viromes were produced by estimating vOTU (length ≥10 kb) numbers along sequencing depth (i.e., read number), which was obtained by subsampling quality-controlled reads (Additional file 2: Fig. S3).

A total of 33 and 107 vOTUs (length ≥10 kb) were obtained for two glacier ice samples (D25 and D49) and the river water control (RiverV) viromes, respectively. Mapping the quality-controlled reads of the 3 viromes to the 140 vOTUs (33+107) showed that the viral communities in the glacier ice samples were completely different from those in the river water control (Additional file 2: Fig. S7), suggesting that the procedures for handling glacier ice samples were “clean,” and no cross contamination was detected among these samples. Only the two glacier ice viromes were used for additional analyses.

The assembled contigs, excluding the predicted viral contigs by VirSorter, were examined for eukaryotic viruses by comparing their genes to the NCBI NR database (non-redundant protein sequence). Only two genes from two contigs (one gene per contig) had significant hits to eukaryotic viruses (bit score 128 and 164). In addition, two other efforts were made to detect eukaryotic viruses (chloroviruses) in the glacier ice samples: (a) the four known chlorovirus hosts, including *Chlorella variabilis* NC64A, *C. variabilis* Syngen 2-3, *C. heliozoae* SAG 3.83, and *Micractinium conductrix* Pbi, were incubated with about 4 ml of melted inner ice water and plaqued for virus [140] and (b) PCR-cloning-sequencing method was used to detect chloroviruses using two pairs of primers mcp F/mep R [141] and CHL Vd F/CHL Vd R [142]. However, none of these experiments detected any chloroviruses. Thus, this study focused on viruses infecting bacteria (bacteriophage).

Taxonomy assignments were performed using vConTACT v2.0 [69]. Briefly, this analysis compared the vOTUs in this study to 2304 viral genomes in the National Center for Biotechnology Information (NCBI) RefSeq database (release v85), and generated VCs approximately equivalent to known viral genera [37, 69, 70]. The putative virus–host linkages were predicted in silico using three methods based on: (i) nucleotide sequence composition, (ii) nucleotide sequence similarity, and (iii) CRISPR spacer matches, as described previously [37, 71]. Thirty-three vOTUs from glacier ice samples were linked to their microbial hosts using the oligonucleotide frequency dissimilarity (VirHostMatcher) measure, with ~32,000 bacterial and archaeal genomes as the host database and a dissimilarity score ≤0.1 and possibility ≥80% as the threshold to pick the host [87]. In addition to sequence composition analysis using VirHostMatcher, the nucleotide sequence of each vOTU was compared (Blastn) to bacterial and archaeal genomes from the NCBI RefSeq database (release v81) and the database (~32,000 genomes) used above. The viral sequences were considered for successful host predictions if they had a bit score of ≥50, E-value of ≤10^−3^, and average nucleotide identity of ≥70% across ≥2,000 bp with the host genomes [37]. Finally, nucleotide sequences of 33 vOTUs were compared to CRISPR spacers of bacterial and archaeal genomes in both databases using the sequence similarity method. The CRISPR spacers with >2 direct repeats in the array were identified using MinCED (mining CRISPRs in environmental data sets [143];) and compared to nucleotide sequences of 33 vOTUs. Hosts were selected if the spacers had zero mismatches to vOTUs.

The putative AMGs were identified and evaluated according to our previously established methods [144]. Specially, all the 33 vOTUs were processed with DRAM-v [145] to obtain gene functional annotations and identify AMGs. Genes on these contigs were regarded as AMGs if having auxiliary scores ≤3 and the M flag. AMGs with transposon regions were not included. To obtain high-quality AMGs, CheckV and manual checking were then used to assess host-virus boundaries and remove the potential host fraction on the viral contig and rule out AMGs potentially coming from microbial contamination using default parameters [146]. Phylogenetic analyses of AMGs were conducted to infer their evolutionary histories. DIAMOND BLASTP [147] was used to query an AMG amino acid sequence against RefSeq database (release v99) in a sensitive mode with default settings, to obtain the reference sequences (top 10 and 100 hits for each viral AMG sequence for conserved motif identification and phylogenetic analysis, respectively). Multiple sequence alignment was performed using MAFFT (v.7.017) [148] with the E-INS-I strategy for 1000 iterations. The aligned sequences were then trimmed using TrimAl [149] with the flag gappyout. The substitution model was selected by ModelFinder [150] for accurate phylogenetic analysis. Phylogenies were generated using IQ-TREE [151] with 1000 bootstrap replicates, and then visualized in iTOL (v5) [152]. Protein sequences from interesting AMGs were structurally modeled using Phyre2 [110] in normal modeling mode to confirm and further resolve functional predictions. The visualization of genome map for the virus containing AMGs of interest was performed using Easyfig version 2.2.5 [153]. Phage genes and hallmark genes were identified by VirSorter [65]. Putative temperate phages were identified by VIBRANT (identified as lysogenic viruses) [154] using its default parameters.

To explore the geographic distribution of glacier viruses, the genome fragments of 33 vOTUs were used as baits to recruit reads from 225 previously published viromes from a wide range of environments including global oceans (145 viromes of GOV 2.0) [66], Arctic sea ice and ancient permafrost brine (cryopeg) [42], soils [72, 73], lakes [74, 75], deserts [76–79], air [80, 81], cryoconite [40], and Greenland ice sheet [40]. The coverage of all vOTUs in each environmental virome was calculated as described above using iVirus BowtieBatch and Read2RefMapper tools [137]. None of the 33 vOTUs were detected from any of these viromes.

### Characterization of phages infecting members of *Methylobacterium*

The 123 previously published viromes (these are the same as the 225 viromes described above, except that the global ocean viromes only included 43 Tara Oceans virome samples [35]) were re-analyzed, by the same method as for glacier-ice viruses, to identify viruses infecting *Methylobacterium*. In addition, *Methylobacterium* viruses (prophages) were also extracted from 131 bacterial genomes within *Methylobacterium* species, which were obtained from the RefSeq database (release v99). These efforts identified 484 *Methylobacterium* phages, which were used for genome-based network analyses to evaluate their relationship with five glacier-ice viruses infecting *Methylobacterium*, using vConTACT version 2 [85, 86]. The genome content and organization for long (>15 kb in size) *Methylobacterium* viruses of interest were evaluated and illustrated by Easyfig version 2.2.5 [153]. The phylogenetic analysis of the DNA circulation protein genes obtained from *Methylobacterium* viruses was performed as described above for AMGs.

## Supplementary Information

**Additional file1: **Table S1 Relative abundance and taxonomy of 33 most abundant OTUs in Inner, Wash and Cut parts of two glacier-ice sections. Table S2 Microbial community composition of eight glacier-ice samples at genus level. Table S3 Physical and chemical characteristics of ice samples from the Guliya ice cap. Table S4 Significance of each parameter's influence on the distribution of microbial communities by Mantel tests. Table S5 General statistics for two viromes. Table S6 Taxonomic assignments and viral cluster information of 33 glacier-ice vOTUs and 2,304 RefSeq genomes. Table S7 Adjusted coverage of 33 vOTUs per gigabase of MetaG for two glacier-ice viromes. Table S8 Predicted hosts taxonomy by sequence similarity method (Blastn). Table S9 Predicted hosts taxonomy by sequence composition method (VirHostMatcher). Table S10 Predicted hosts taxonomy by CRISPR similarity method. Table S11 Temperate viruses predicted by VIBRANT from the 33 vOTUs. Table S12 *Methylobacterium* viruses identified from glacier ice, bacterial genomes in RefSeq database and environmental metagenomes. Table S13 Annotations of all genes from 33 vOTUs. Table S14 Four AMGs identified from glacier-ice viruses. Table S15 Annotations of 70 viral contigs’ genes from the Global Ocean Viromes 2.0 dataset.

**Additional file2: **Figure S1 Ice core sampling and preparation in the laboratory. (a) The cold work room (−5°C) with band saw, BioGard laminar flow hood and wash systems. (b) the outer layer of the ice section being removed by the band saw. (c) The ice section being washed with 95% ethanol and (d) with water. (e) The “clean” inner ice is preserved in the autoclaved beakers or bottles. Figure S2 Microbial communities at genus level (a) and overlapped OTUs (b) of removed and inner ice samples collected during decontamination procedures. The most abundant genera (n = 30) and OTUs (n = 33) are illustrated. Cut, Wash and Inner represent ice samples collected from band saw scrapping, water washing and the inner ice, respectively. Figure S3 Rarefaction curves of two glacier-ice viromes by vOTU numbers. Rarefaction curves were constructed by the change of vOTUs (≥10 kb) number along sequencing depth (i.e., read number) obtained by subsampling quality-controlled reads. Figure S4 The unrooted neighbor-joining phylogenetic tree of Mu *N* genes from eight *Methylobacterium* viruses. The tree was constructed using the predicted amino acid sequences of the *N* genes from two glacier ice viruses (i.e., D25_14_65719 and D49_170_39214; in bold font) and six prophages identified from bacterial genomes. Each viral contig contains two copies of *N* genes. Viruses belonged to the same VC (i.e., VC0_0 or VC8_0) are indicated in the same color. Bootstrap values (expressed as percentages of 1,000 replications) are shown at the branch points. The scale bar indicates a distance of 0.2. Figure S5 Characterization of virus-encoded auxiliary metabolic genes (AMGs). (a) Genome map of glacier-ice virus D25_22_20338 encoding AMGs (motility genes *motA* and *motB*). CheckV was used to assess host-virus boundaries and remove potential host fractions on the viral contig (See Materials and Methods). Genes were marked by four colors to illustrate AMGs (red), phage genes (orange), potential cellular genes (green), and unaffiliated genes (grey). AMGs were detected by DRAM-v and following manual inspection; The latter three groups of genes were classified by comparing their predicted protein sequences to those of a large database of 15,958 profile hidden Markov models by CheckV and of viral genes in the extended RefSeqABVir database by VirSorter v1 in virome decontamination mode. Genes were marked as “phage genes” if they were matched to the genes of viruses in RefSeqABVir database or CheckV databases. Genes were marked as “potential cellular genes” if they were matched to the genes of bacteria or archaea by CheckV. Genes were considered “unaffiliated” if they had no hit to a sequence in RefSeqABVir or CheckV databases. (b-c) Predicted three-dimensional (3D) structures of AMG products and templates. The 3D structure of template protein for each AMG is at the right (i.e., c6ykmB and v3ckhnB). Both AMG products are linked to their closest template protein with 100% confidence score by phyre2. (d-e) Multiple alignments of protein sequences for two AMGs and 10 closest related bacteria-originated genes. The AMG and 10 closest related bacteria-related genes are numbered as 1 and 2-11, respectively. Conserved motif of the MotB was indicated by black boxes and notes (i.e., conserved peptidoglycan-binding motif). MotA does not have a conserved motif. ‘h’ indicates hydrophobic amino acid and ‘x’ indicates any amino acid. The protein sequences were aligned using MAFFT (v.7.017) with the E-INS-I strategy for 1000 iteration. The position numbers of aligned sequences are indicated at the top. Figure S6 Phylogenetic analysis of two novel AMG products MotA (A) and MotB (B). Phylogenetic trees are inferred using maximum likelihood method with amino acid sequences (see Materials and Methods). The genes from glacier-ice virus (i.e., AMGs) and the NCBI RefSeq database (release v99) are colored in red and black, respectively. The scale bars indicate a distance of 0.1. Bootstrap values (expressed as percentages of 1000 replications) ≥50 are shown at the branch points. Figure S7 Heatmap showing the viral community compositions of two glacier-ice and one river-water viromes. Glacier ice samples: D25 and D49; River water sample: RiverV. The coverages of 140 vOTUs (>10 kb; 33 and 107 vOTUs from glacier ice and river water, respectively) were normalized to per gigabase of metagenome.

## Data Availability

The amplicon sequences obtained in this study have been deposited in the NCBI Sequence Read Archive under BioProject accession number PRJNA594142. The viral metagenomes are available through iVirus (https://datacommons.cyverse.org/browse/iplant/home/shared/iVirus/Tibet_Glacier_viromes_2017), including raw and quality-controlled reads and vOTUs.

## Abbreviations

vOTU: Viral operational taxonomic unit
VLP: Virus-like particle
PCoA: Principal Coordinates Analysis
ANOSIM: Analysis of similarity statistics
VC: Viral cluster
CBA 18: *Cellulophaga baltica* strain #18
PS core: Plateau shallow core
qPCR: Quantitative polymerase chain reaction
OTU: Operational taxonomic unit
RDP: Ribosomal database project
