# Supplementary material for: Glacier ice archives nearly 15,000-year-old microbes and phages
Source: Microbiome. 2021 Jul 20;9:160. doi: 10.1186/s40168-021-01106-w (PMC8290583; doi:10.1186/s40168-021-01106-w)
Supplement: Supplementary file 3 — Additional file2: Figure S1 Ice core sampling and preparation in the laboratory. (a) The cold work room (−5°C) with band saw, BioGard laminar flow hood and wash systems. (b) the outer layer of the ice section being removed by the band saw. (c) The ice section being washed with 95% ethanol and (d) with water. (e) The “clean” inner ice is preserved in the autoclaved beakers or bottles. Figure S2 Microbial communities at genus level (a) and overlapped OTUs (b) of removed and inner ice samples collected during decontamination procedures. The most abundant genera (n = 30) and OTUs (n = 33) are illustrated. Cut, Wash and Inner represent ice samples collected from band saw scrapping, water washing and the inner ice, respectively. Figure S3 Rarefaction curves of two glacier-ice viromes by vOTU numbers. Rarefaction curves were constructed by the change of vOTUs (≥10 kb) number along sequencing depth (i.e., read number) obtained by subsampling quality-controlled reads. Figure S4 The unrooted neighbor-joining phylogenetic tree of Mu N genes from eight Methylobacterium viruses. The tree was constructed using the predicted amino acid sequences of the N genes from two glacier ice viruses (i.e., D25_14_65719 and D49_170_39214; in bold font) and six prophages identified from bacterial genomes. Each viral contig contains two copies of N genes. Viruses belonged to the same VC (i.e., VC0_0 or VC8_0) are indicated in the same color. Bootstrap values (expressed as percentages of 1,000 replications) are shown at the branch points. The scale bar indicates a distance of 0.2. Figure S5 Characterization of virus-encoded auxiliary metabolic genes (AMGs). (a) Genome map of glacier-ice virus D25_22_20338 encoding AMGs (motility genes motA and motB). CheckV was used to assess host-virus boundaries and remove potential host fractions on the viral contig (See Materials and Methods). Genes were marked by four colors to illustrate AMGs (red), phage genes (orange), potential cellular genes (green), a [file 40168_2021_1106_MOESM2_ESM.docx]

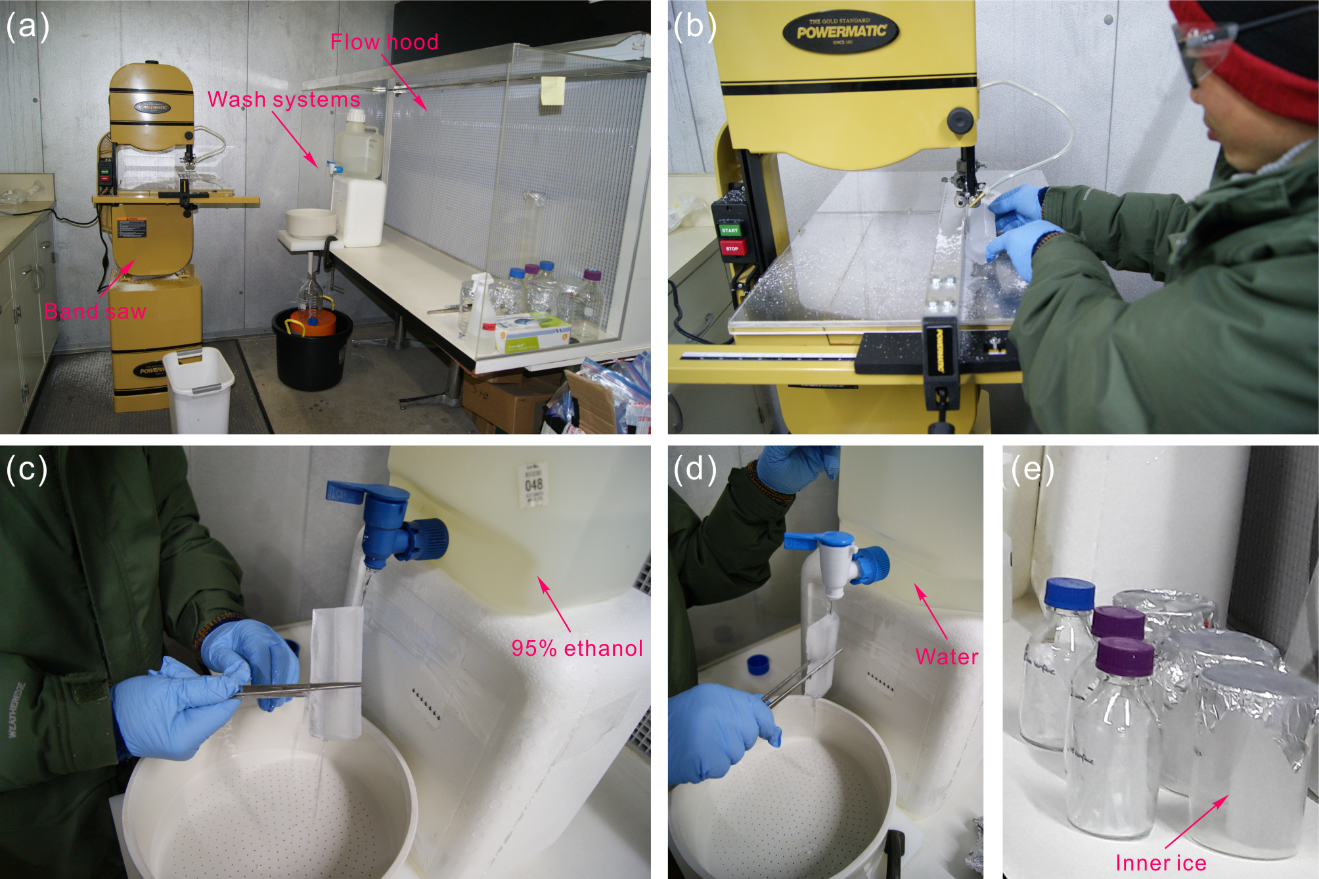


**Supplementary Figure S1 Ice core sampling and preparation in the laboratory.** (**a**) The cold work room (−5ºC) with band saw, BioGard laminar flow hood and wash systems. (**b**) the outer layer of the ice section being removed by the band saw. (**c**) The ice section being washed with 95% ethanol and (**d**) with water. (**e**) The “clean” inner ice is preserved in the autoclaved beakers or bottles.


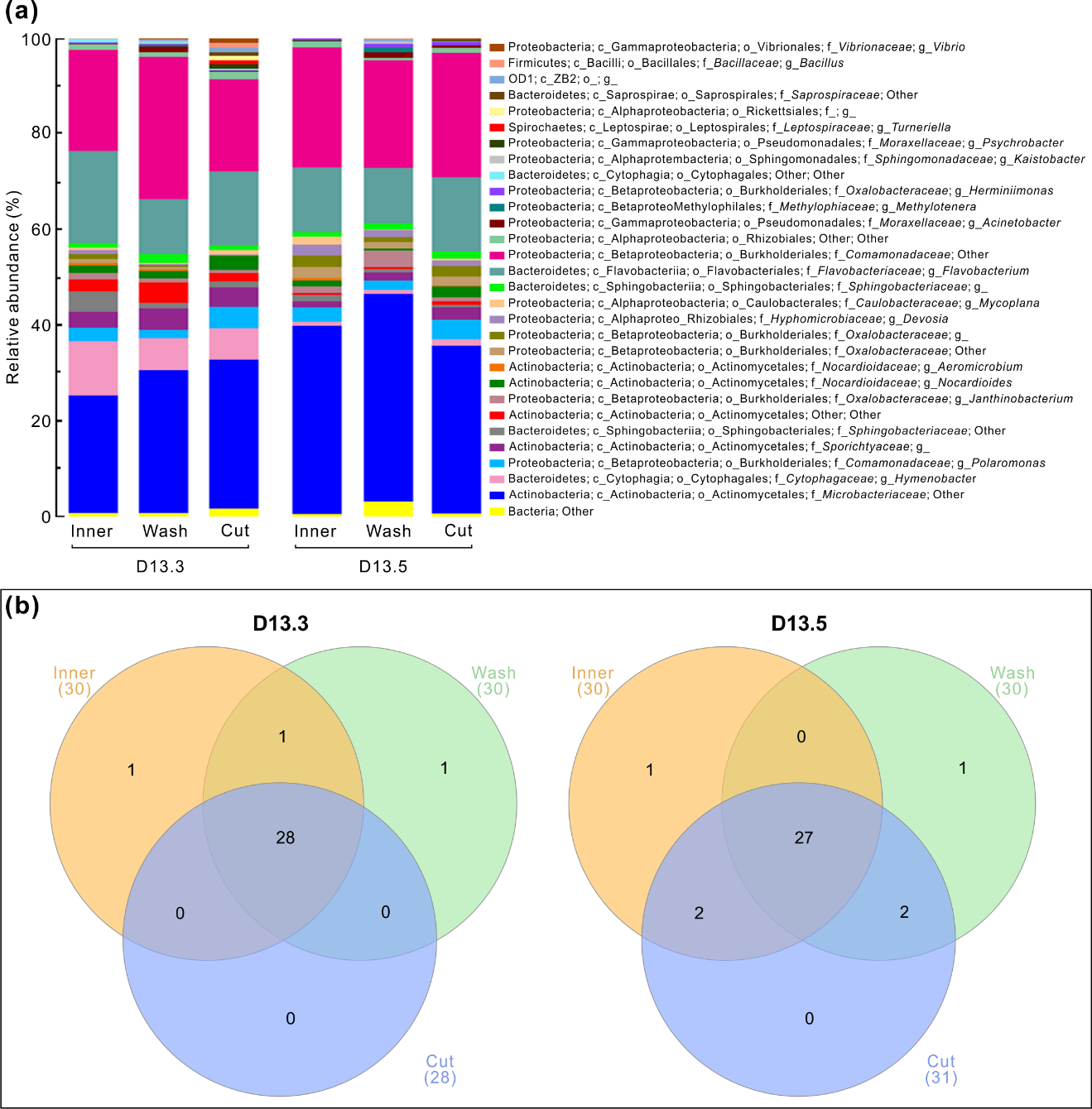


**Supplementary Figure S2 Microbial communities at genus level (a) and overlapped OTUs (b) of removed and inner ice samples collected during decontamination procedures.** The most abundant genera (n = 30) and OTUs (n = 33) are illustrated. Cut, Wash and Inner represent ice samples collected from band saw scrapping, water washing and the inner ice, respectively.

**
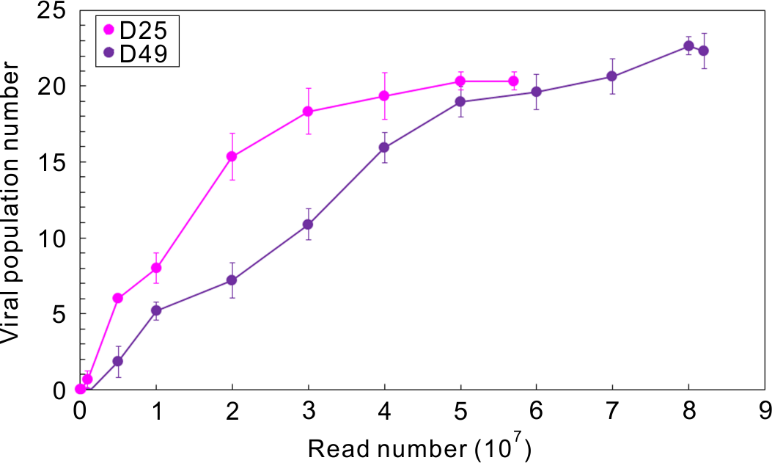
**

**Supplementary Figure S3 Rarefaction curves of two glacier-ice viromes by vOTU numbers.** Rarefaction curves were constructed by the change of vOTUs (≥10 kb) number along sequencing depth (i.e., read number) obtained by subsampling quality-controlled reads.

**
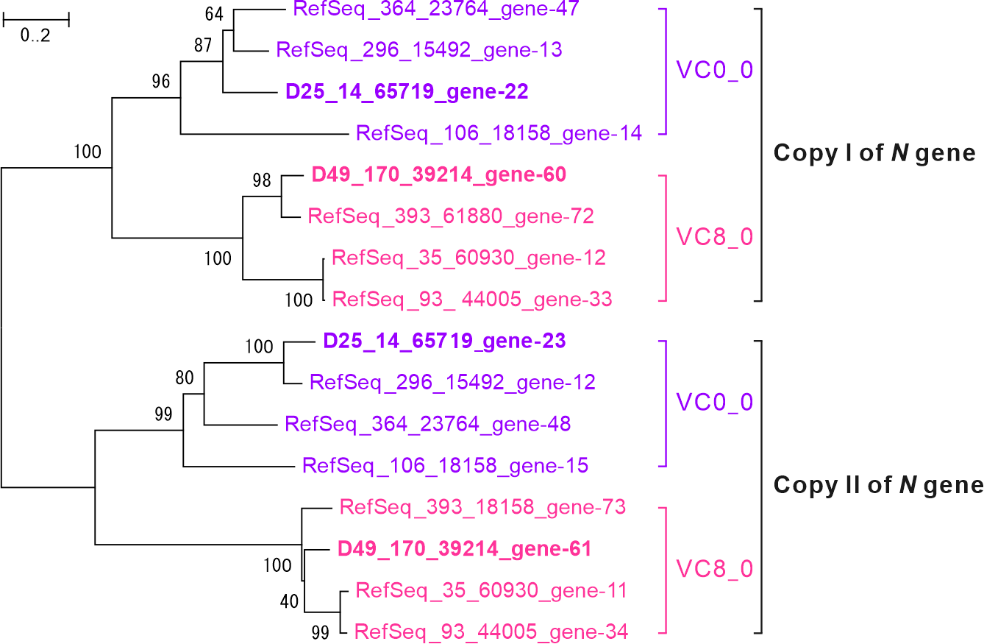
**

**Supplementary Figure S4 The unrooted neighbor-joining phylogenetic tree of Mu *N* genes from eight *Methylobacterium* viruses.** The tree was constructed using the predicted amino acid sequences of the *N* genes from two glacier ice viruses (i.e., D25_14_65719 and D49_170_39214; in bold font) and six prophages identified from bacterial genomes. Each viral contig contains two copies of *N* genes. Viruses belonged to the same VC (i.e., VC0_0 or VC8_0) are indicated in the same color. Bootstrap values (expressed as percentages of 1,000 replications) are shown at the branch points. The scale bar indicates a distance of 0.2.

**
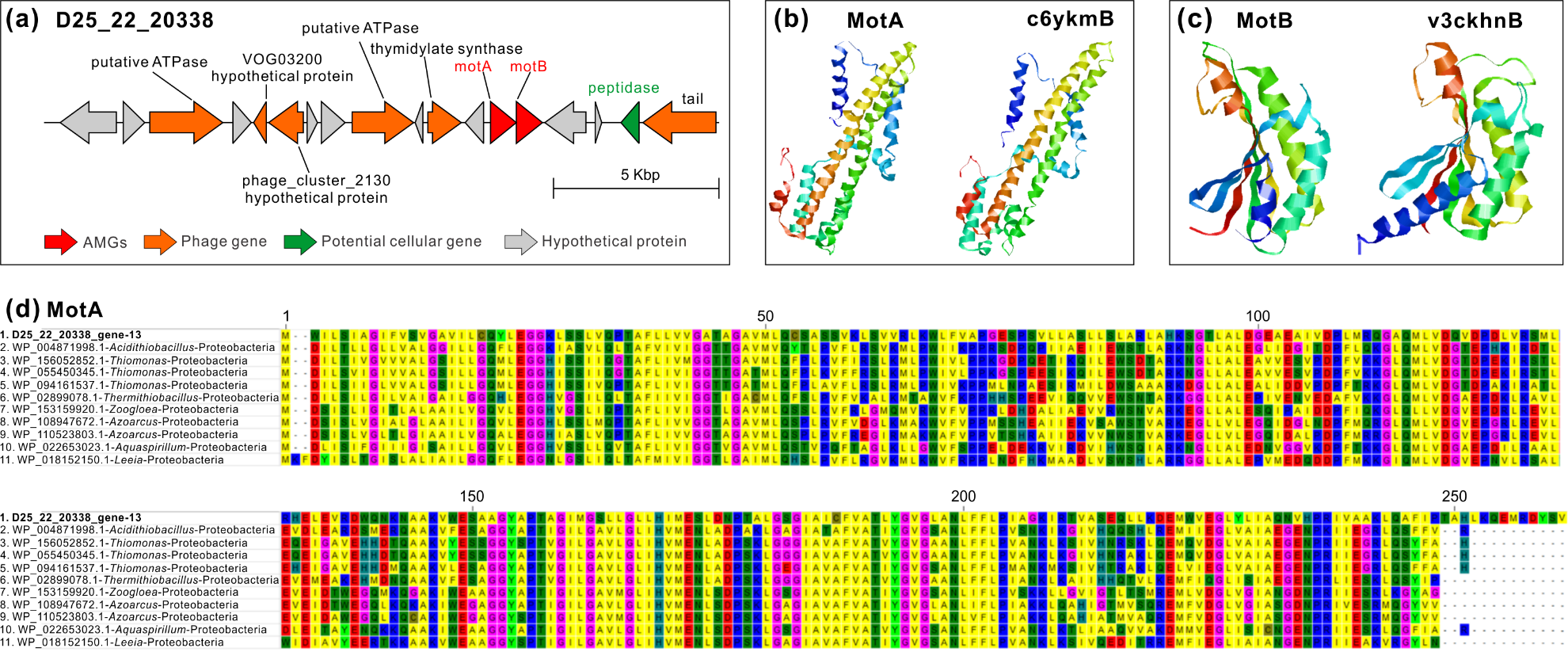
**

**
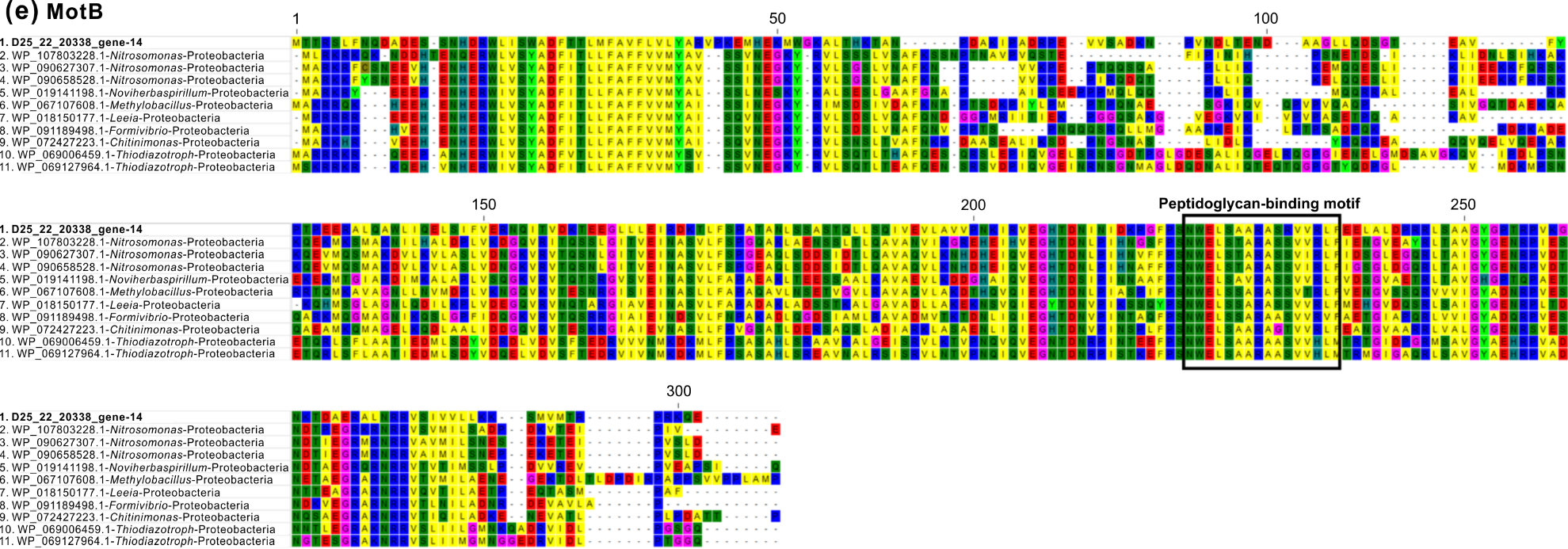
**

**Supplementary Figure S5 Characterization of virus-encoded auxiliary metabolic genes (AMGs).** (**a**) Genome map of glacier-ice virus D25_22_20338 encoding AMGs (motility genes *motA* and *motB*). CheckV was used to assess host-virus boundaries and remove potential host fractions on the viral contig (See Materials and Methods). Genes were marked by four colors to illustrate AMGs (red), phage genes (orange), potential cellular genes (green), and unaffiliated genes (grey). AMGs were detected by DRAM-v and following manual inspection; The latter three groups of genes were classified by comparing their predicted protein sequences to those of a large database of 15,958 profile hidden Markov models by CheckV and of viral genes in the extended RefSeqABVir database by VirSorter v1 in virome decontamination mode. Genes were marked as “phage genes” if they were matched to the genes of viruses in RefSeqABVir database or CheckV databases. Genes were marked as “potential cellular genes” if they were matched to the genes of bacteria or archaea by CheckV. Genes were considered “unaffiliated” if they had no hit to a sequence in RefSeqABVir or CheckV databases. (**b-c**) Predicted three-dimensional (3D) structures of AMG products and templates. The 3D structure of template protein for each AMG is at the right (i.e., c6ykmB and v3ckhnB). Both AMG products are linked to their closest template protein with 100% confidence score by phyre2. (**d-e**) Multiple alignments of protein sequences for two AMGs and 10 closest related bacteria-originated genes. The AMG and 10 closest related bacteria-related genes are numbered as 1 and 2-11, respectively. Conserved motif of the MotB was indicated by black boxes and notes (i.e., conserved peptidoglycan-binding motif). MotA does not have a conserved motif. ‘h’ indicates hydrophobic amino acid and ‘x’ indicates any amino acid. The protein sequences were aligned using MAFFT (v.7.017) with the E-INS-I strategy for 1000 iteration. The position numbers of aligned sequences are indicated at the top.

**
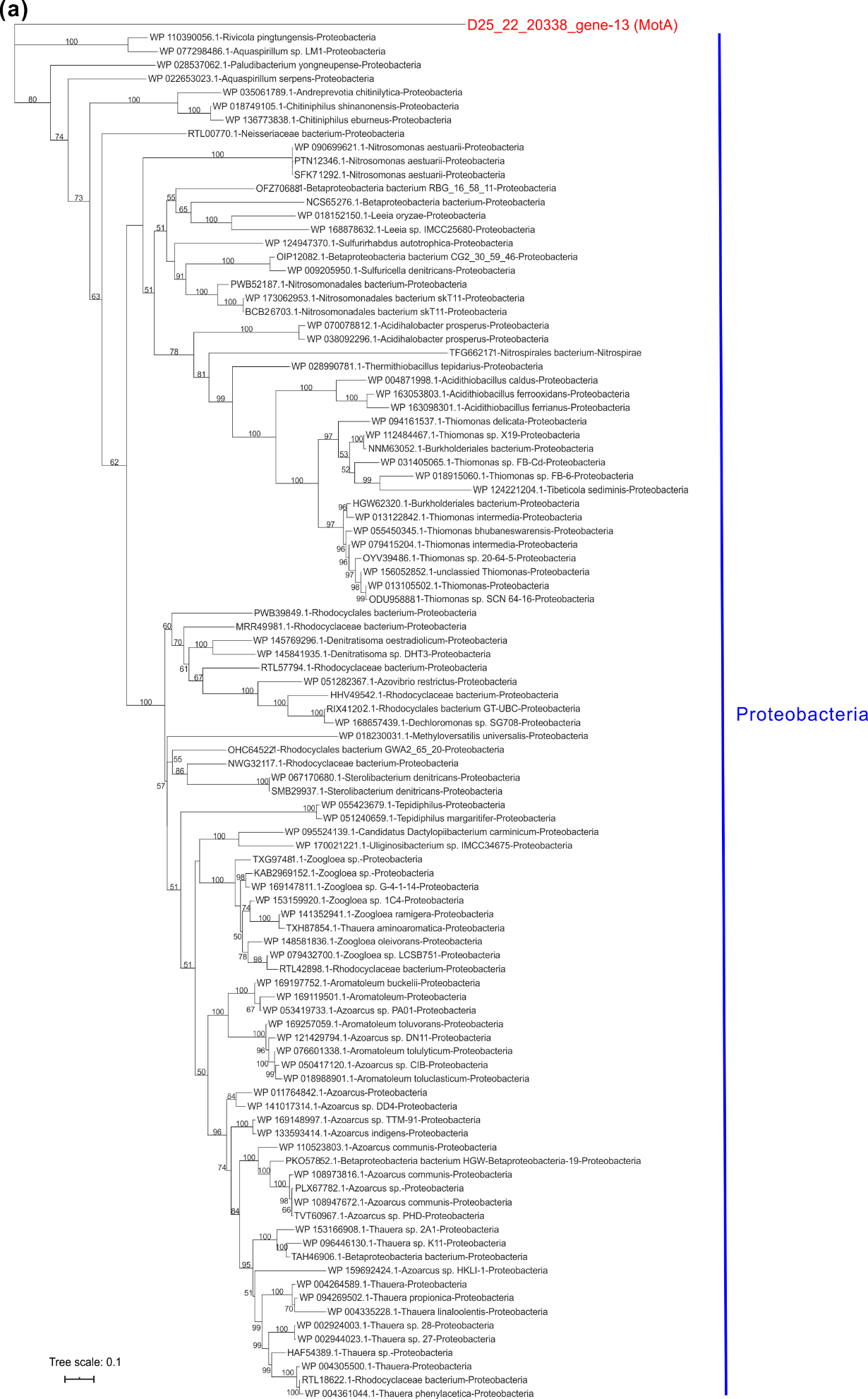
**

**
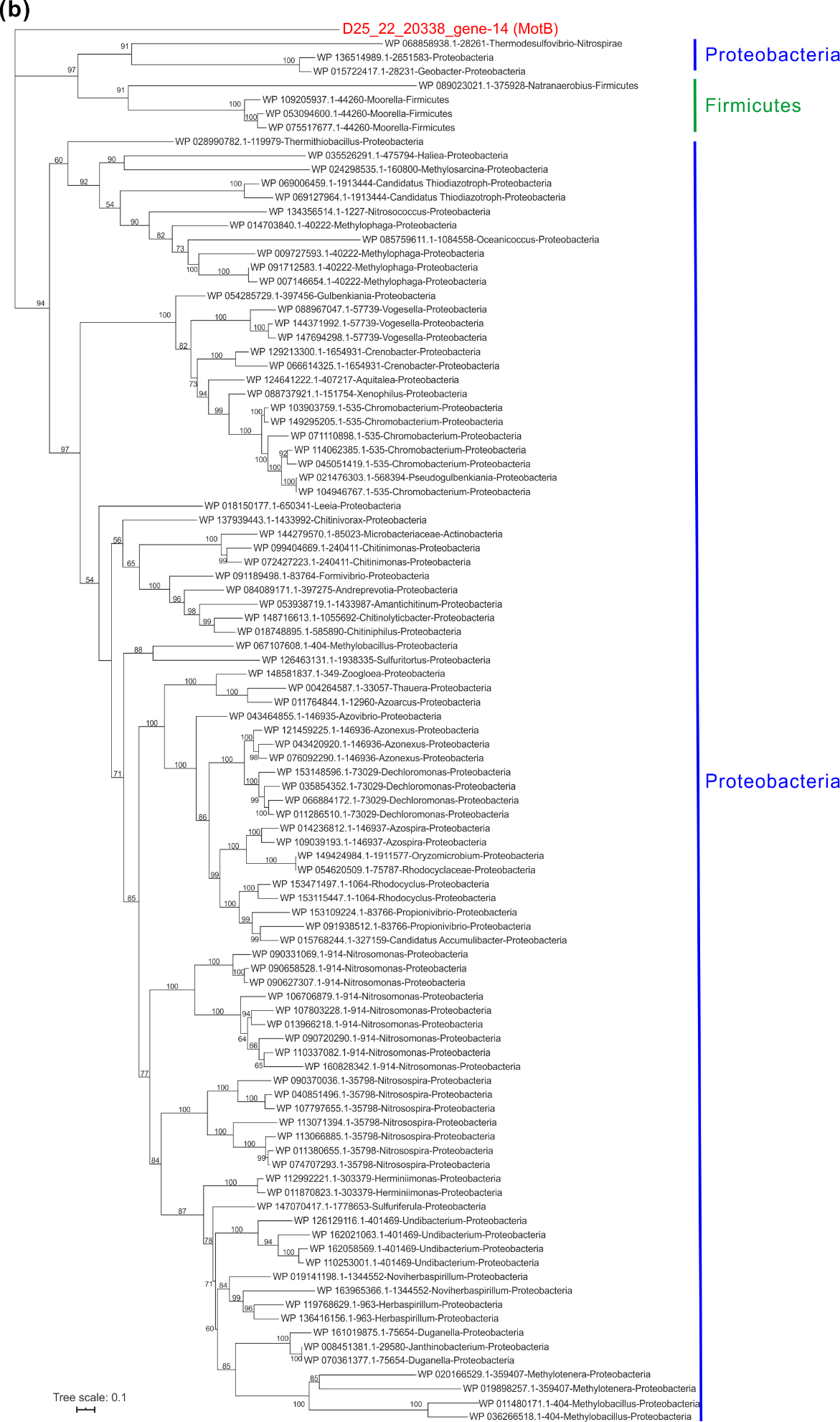
**

**Supplementary Figure S6 Phylogenetic analysis of two novel AMG products MotA (A) and MotB (B).** Phylogenetic trees are inferred using maximum likelihood method with amino acid sequences (see Materials and Methods). The genes from glacier-ice virus (i.e., AMGs) and the NCBI RefSeq database (release v99) are colored in red and black, respectively. The scale bars indicate a distance of 0.1. Bootstrap values (expressed as percentages of 1000 replications) ≥50 are shown at the branch points.

**
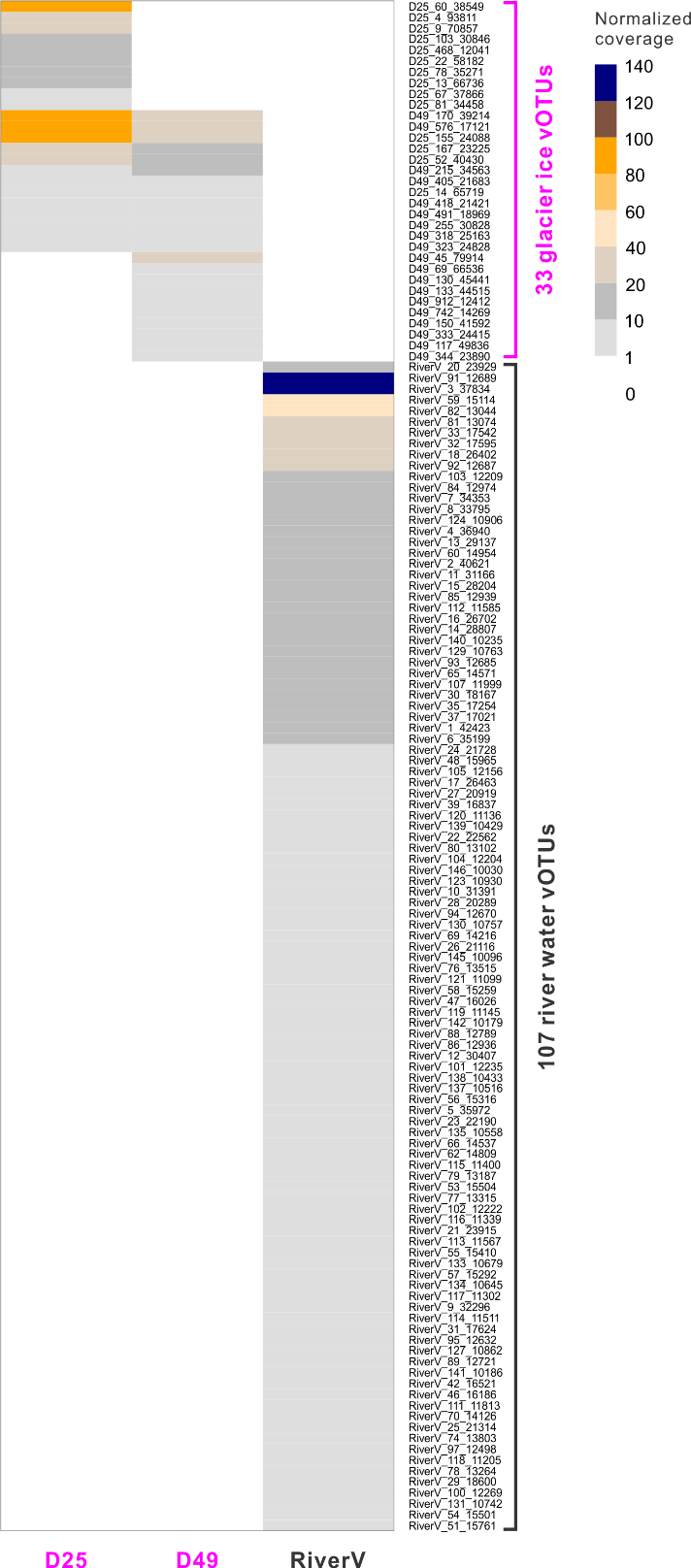
**

**Supplementary Figure S7 Heatmap showing the viral community compositions of two glacier-ice and one river-water viromes.** Glacier ice samples: D25 and D49; River water sample: RiverV. The coverages of 140 vOTUs (>10 kb; 33 and 107 vOTUs from glacier ice and river water, respectively) were normalized to per gigabase of metagenome.
